# Circadian gene signatures in the progression of obesity based on machine learning and Mendelian randomization analysis

**DOI:** 10.3389/fnut.2024.1407265

**Published:** 2024-09-16

**Authors:** Zhi’ang Cheng, Binghong Liu, Xiaoyong Liu

**Affiliations:** ^1^Department of Ophthalmology, The First Affiliated Hospital of Jinan University, Jinan University, Guangzhou, China; ^2^College of Horticulture, South China Agricultural University, Guangzhou, China; ^3^Department of Ophthalmology, The Affiliated Shunde Hospital of Jinan University, Foshan, China

**Keywords:** obesity, circadian gene, immune, molecular mechanism, Mendelian randomization, machine learning

## Abstract

**Objective:**

Obesity, a global health concern, is associated with a spectrum of chronic diseases and cancers. Our research sheds light on the regulatory role of circadian genes in obesity progression, providing insight into the immune landscape of obese patients, and introducing new avenues for therapeutic interventions.

**Methods:**

Expression files of multiple datasets were retrieved from the GEO database. By 80 machine-learning algorithm combinations and Mendelian randomization analysis, we discovered the key circadian genes contributing to and protecting against obesity. Subsequently, an immune infiltration analysis was conducted to examine the alterations in immune cell types and their abundance in the body and to investigate the relationships between circadian genes and immune cells. Furthermore, we delved into the molecular mechanisms of key genes implicated in obesity.

**Results:**

Our study identified three key circadian genes (BHLHE40, PPP1CB, and CSNK1E) associated with obesity. BHLHE40 was found to promote obesity through various pathways, while PPP1CB and CSNK1E counteracted lipid metabolism disorders, and modulated cytokines, immune receptors, T cells, and monocytes.

**Conclusion:**

In conclusion, the key circadian genes (BHLHE40, CSNK1E, and PPP1CB) may serve as novel biomarkers for understanding obesity pathogenesis and have significant correlations with infiltrating immune cells, thus providing potential new targets for obese prevention and treatment.

## 1 Introduction

Obesity, a pressing global health issue, is associated with numerous complications such as type 2 diabetes, cardiovascular disease, obstructive sleep apnea, and various cancers, making it a leading cause of increasing mortality worldwide (1). Current studies highlight that obesity is a complex disease with a multifaceted etiology, encompassing genetic, metabolic, behavioral, and environmental factors (2). Firstly, an energy imbalance caused by caloric intake surpassing expenditure is a pivotal factor in developing obesity. Redundant energy is stored as fat, leading to pathological obesity when it significantly exceeds the body’s energy utilization (3). Secondly, lifestyles and environmental factors play crucial roles in weight gain. The decrease in leisure time and physical activities, the rise in sedentary behaviors (such as electronic device usage), and sleep deprivation caused by chronic work stress collectively contribute to the concurrent occurrence of chronic diseases and obesity (2). Thirdly, genetic research has demonstrated a polygenic mechanism for obesity susceptibility, with the FTO gene variant being the most notable. Large-scale genomic screenings have shown that individuals carrying one or two copies of this risk allele gain approximately a body weight of 1.2 to 3 kg more than non-carriers (4). Additionally, depression and anxiety significantly increase the risk of obesity (5). The association of obesity with multiple diseases underscores the importance of its treatment and management in clinical practice.

Almost all mammals have developed a self-regulated transcription-translation feedback loop, generating approximately 24 h oscillations known as the biological clock. The primary function of the biological clock is to regulate the circadian rhythm of physiological processes, ensuring synchronization with the external environment (6). Disruptions in circadian rhythm, caused by genetic or environmental factors, can have long-term detrimental effects on metabolic health (7). The reciprocal regulation of circadian rhythm and lipid metabolism is increasingly recognized as a critical study area. The interaction between the biological clock and lipid metabolism may play a role in the occurrence and development of obesity. Recent studies suggest that various nutritional sensors can transmit information about nutritional status to the biological clock, indicating a bidirectional relationship. For example, early research demonstrated that reduced nicotinamide adenine dinucleotide (NADH) increases the activity of core clock genes (CLOCK/BMAL1 and NPAS2/BMAL1), while nicotinamide adenine dinucleotide (NAD) inhibits their activity (8). AMP kinase (AMPK), another highly conserved cellular nutrition sensor, is activated by exercise, fasting, or hypoxia, leading to the phosphorylation and degradation of CRY1 (9, 69). Additionally, metabolic transcription factors such as REV-ERB, ROR, and PPAR exhibit circadian expression in peripheral tissues (10). REV-ERBα regulates adipocyte differentiation and inhibits the transcription of BMAL1 (11). RORα competes with REV-ERBα to bind to the BMAL1 promoter, inducing BMAL1 expression and regulating lipid metabolism (12). PPARα positively regulates BMAL1 expression, creating a positive feedback loop (13, 70). Besides, genetic variations in CLOCK, PER2, and CRY1 increase the risk of obesity (14). This indicates complex crosstalk between circadian genes and transcriptional regulatory factors, though the specific mechanisms by which circadian genes directly participate in obesity remain unclear.

With the accelerated advancements in bioinformatics in recent years, techniques such as microarray sequencing, transcriptome sequencing, and single-cell sequencing have become instrumental in identifying novel mechanisms underlying obesity pathogenesis and discovering new biomarkers (15, 71). The development of bioinformatics technology has enabled researchers to discover that cytokines such as macrophages and IL-17 are involved in the development of obesity, providing a more comprehensive understanding of the molecular changes in this disease (16, 72). Simultaneously, numerous large-scale genome-wide association studies (GWAS) have identified a significant number of single nucleotide polymorphisms (SNPs) associated with obesity risk. Mendelian randomization (MR) is a genetic analytical method that operates under Mendel’s laws of inheritance, using SNPs as instrumental variables (IVs) to establish observed causal correlations between modifiable exposures and clinical outcomes (17). By combining pooled data from disease GWAS and expression quantitative trait loci (eQTL) studies, MR analyses have been widely used in studying multiple diseases, elucidating causal correlations between genes and diseases, and discovering new therapeutic targets. In MR analyses, the expression level of a gene is considered an exposure, while eQTLs situated in genomic regions can represent genes in that genome segment. The reliability and robustness of these results are subsequently assessed by various rigorous statistical procedures (18, 73). Therefore, MR based on GWAS is crucial in providing accurate and direct evidence of pathogenic genes and drug targets.

In this study, we applied 10 machine learning algorithms to identify characteristic genes and subsequently used MR analysis to pinpoint circadian genes related to obesity. Besides, this study was supplemented by examining the molecular mechanisms and the immune landscape underlying obesity. We explored the immune infiltration in obesity and analyzed the correlations between key genes and immune cells by immune infiltration analysis. Finally, enrichment analysis was conducted to uncover the possible functional mechanisms of these key genes and the effect of immune inflammation in the pathogenesis.

## 2 Experimental methods

### 2.1 Materials and methods

#### 2.1.1 Study design and data acquisition

**Figure 1:**
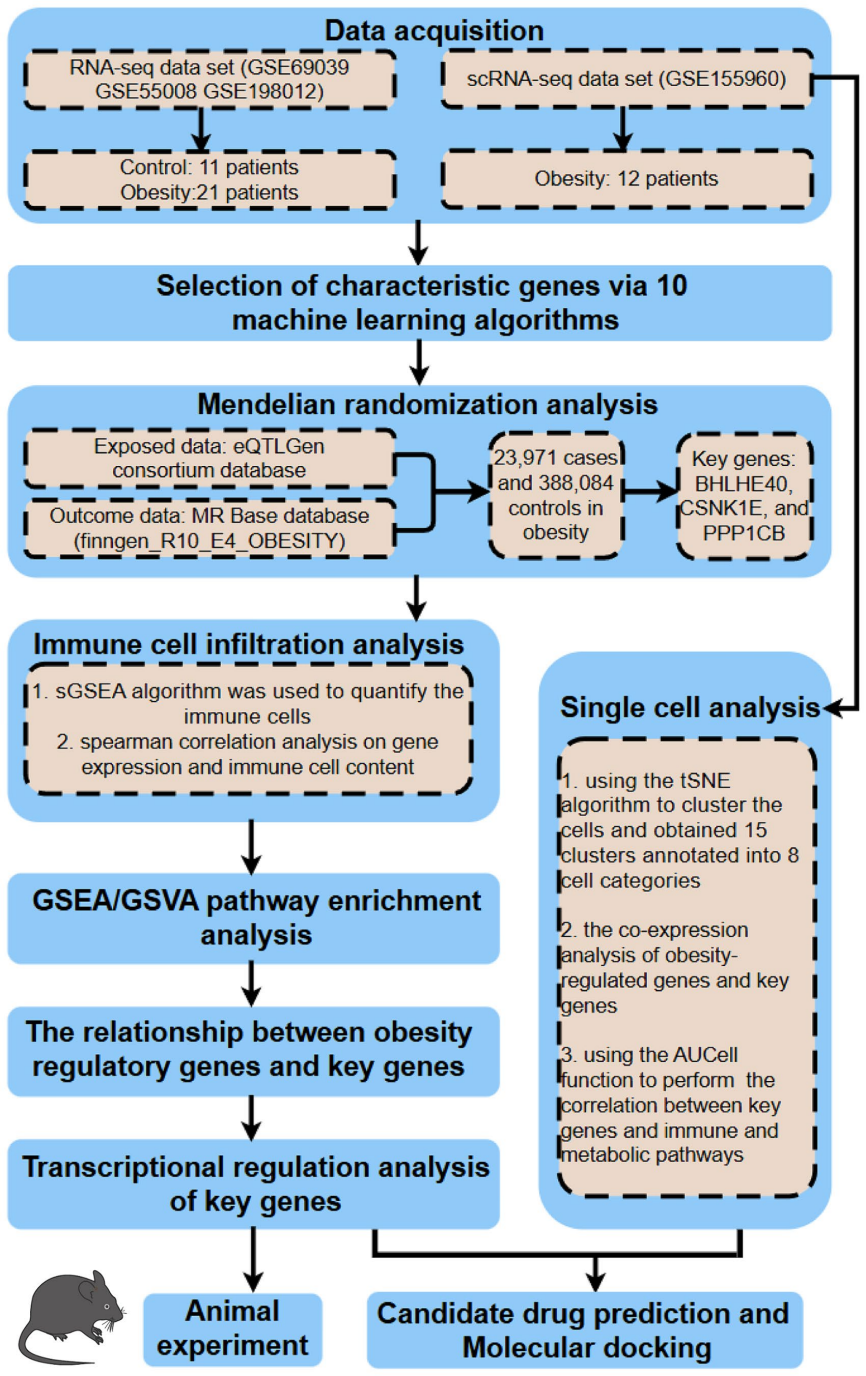
Flowchart of study design.

The analysis process of this bioinformatics research is illustrated in Figure 1. For our study, we downloaded the expression files of GSE69039 from the GEO database (Gene Expression Omnibus, https://www.ncbi.nlm.nih.gov/geo/), annotated with GPL10558. This dataset includes expression profile data from 18 samples (4 controls and 14 patients). Additionally, we obtained the rawcounts files of GSE55008 and GSE198012 and transformed the gene IDs into symbols using the R package “org.Hs.eg.db.” We also downloaded the single-cell dataset of GSE155960, including 12 patients with complete expression profiles for analysis. By exclusively selecting data from human subjects, we eliminated interspecies differences, enhancing the reliability of our study. Compared to previous research, more comprehensive datasets strengthen the robustness of our findings. The patients included in this study were diagnosed with obesity without any other metabolic, or psychiatric disorders. Furthermore, we did not classify obesity by subtypes and did not consider differences in race, region, or gender to eliminate potential biases that could affect the results. To address non-biological effects caused by different samples, sequencing technologies, and instruments between microarray and RNA-seq data, we integrated and analyzed multiple datasets using the Rank-in algorithm (19).

eQTL data from the eQTLGen database^1^ was extracted as the exposure. The large-scale eQTLGen project focuses on conducting large-scale genome-wide meta-analyses in blood and investigating the genetic basis of complex traits. Participants in the outcome-related GWAS studies for this study were primarily European. The outcome data were all derived from the MR Base database (finngen_R10_E4_OBESITY) and included 23,971 patients and 388,084 controls. It contains publications, top associations, and complete summary statistics, which could be mapped to genome assembly and dbSNP build.

#### 2.1.2 Selection of characteristic genes via machine learning

Our study involved binary data, and ten machine learning algorithms were selected based on sample size, quality, and the research question. To identify the key genes involved in obesity, we followed the previous workflow (20). Due to the researchers’ focus on tumor prognosis, we have improved several classic machine learning algorithms to better fit the binary classification problem in this study. (1) Firstly, we integrated 10 classic algorithms: random forest (RF), gradient booster machine (GBM), support vector machine (SVM), logistic regression (LR), Lasso, Ridge, XGBoost, Naive Bayes, Stepwise Cox, and Elastic Network (Enet). Among them, RF, LASSO, XGBoost, and Stepwise Cox have the function of dimensionality reduction and variable screening, and we combined them with other algorithms into 80 machine-learning algorithm combinations. (2) Next, we used GSE69039 with a large sample size in GEO as the training cohort and used these 80 combinations to construct signatures in the files with different expression genes (DEGs). (3) Finally, in the two testing cohorts (GSE55008, GSE198012), we calculated the Harrell’s concordance index (C-index) for model selection. Based on the average C-index, we ultimately chose the best consensus prediction model for obesity and obtained the average ranks of each signature. Signatures with model number >10 and average rank <6 were defined as characteristic genes. In addition, external independent datasets (GSE241015, GSE231656, GSE133666) were generated to evaluate the diagnostic performance of the optimal model through receiver operating characteristic (ROC) curves. We utilized the Caret package to fine-tune and train each algorithm meticulously to achieve optimal performance, identifying the algorithm with the highest accuracy and diagnostic efficiency.

#### 2.1.3 Mendelian randomization analysis

The outcome IDs filtered through the MR-Base database^2^ are derived from the GWAS summary data^3^ to extract relevant causal correlations in eQTL. Firstly, we conducted a preliminary screening of basic information to investigate the causal relationship between circadian rhythm and obesity. SNPs were screened as IVs based on the following criteria: (1) SNPs demonstrated a significant genome-wide correlation (*p* < 5 × 10^−8^) in the initial analysis, indicating a strong association between SNPs and exposure; (2) linkage disequilibrium (LD) between SNPs was excluded (*R*^2^ < 0.001 and <10,000 kb) in the sensitivity analysis; (3) the *F*-value was used to assess the strength of IVs for screened SNPs to avoid weak instrumental bias. Generally, an *F*-value of 10 or higher indicates that the IVs strongly correlate with exposure and that MR results are not influenced by weak instrumental bias. Secondly, we removed palindromic SNPs with moderate allele frequencies and retained SNPs with *p* < 5 × 10^−5^. The selected SNPs were then examined for their association with potential confounding factors such as diabetes and hormone use in Phenoscanner^4^. Finally, we conducted MR (21) analysis to establish causal relationships using genetic variations as IVs for exposure (circadian genes).

MR analysis is based on three core assumptions: (1) the correlation assumption (IVs are closely related to the exposure but not directly related to the outcomes); (2) the independence assumption (IVs are not related to confounding factors); and (3) the exclusivity assumption (IVs affect outcomes only through the exposure; any effect through other pathways indicates genetic pleiotropy). We primarily used the inverse variance weighted (IVW) method for MR analysis, which provides a consistent estimate of the association between exposure and outcome risk without pleiotropy (22). The IVW and MR Egger tests were then performed to evaluate heterogeneity among individual SNPs. A *p*-value less than 0.05 indicates no heterogeneity, suggesting that fixed-effect models should be used. Otherwise, random-effect models are appropriate (23). Additionally, we conducted a pleiotropy analysis to verify the robustness of our results. The MR Egger intercept method was used to determine whether IVs exhibited pleiotropy. When the intercept term is very close to zero, MR Egger regression is nearly equivalent to IVW. A lower likelihood of horizontal pleiotropy indicates that SNPs are associated only with the exposure and not with other confounding variables (24). The weighted mode is used to correct for pleiotropy and is more reliable in detecting causal effects, with smaller bias and lower Type I error rates than the MR Egger test. When measurement errors occur in the exposure of SNPs, the weighted median allows for accurate estimation of causal relationships, even when up to 50% of IVs are invalid, thereby reducing bias in the estimation of causal correlations (25).

The reliability of causal relationships was evaluated using four methods to obtain an overall estimate of the impact of cis- and trans-regional gene expressions in whole blood on obesity. Finally, we conducted a sensitivity analysis to assess the impact of specific genetic variations on the risk of obesity. This method systematically excludes each SNP and recalculates the combined effects of the remaining SNPs to identify and eliminate those variations that significantly impact the overall estimated value. The removal of each SNP results in a new point estimate and its 95% confidence interval, allowing for the evaluation of its unique contribution and the robustness of the overall results. This analysis produces a chart summarizing the estimated values after the removal of each SNP, as well as the overall estimated values of all SNPs. By comparing these estimates, we can observe the impact of removing any single SNP on the overall results, thereby determining the robustness of our analysis.

#### 2.1.4 Immune cell infiltration analysis

The single sample gene set enrichment analysis (ssGSEA) is widely used to evaluate immune cell types in the microenvironment. This method distinguishes 29 human immune cell phenotypes, including T cells, B cells, and NK cells. In this study, the ssGSEA algorithm was utilized to quantify the immune cells in the expression profile, infer the relative proportions of 29 types of immune infiltrating cells, and perform Spearman correlation analysis on gene expression and content of immune cells.

#### 2.1.5 GSEA pathway enrichment analysis

Gene Set Enrichment Analysis (GSEA) analysis is frequently employed in research that closely integrates disease classification with biological significance. GSEA was used to further analyze the differences in signaling pathways between high- and low-expression groups. The background gene sets were version 7.0, downloaded from the Molecular Signatures Database (MsigDB, https://www.gsea-msigdb.org/gsea/msigdb/). These annotated gene sets for subtype pathways enabled the differential expression analysis of pathways between subtypes. Significantly enriched gene sets were sorted based on an adjusted *p*-value of less than 0.05.

#### 2.1.6 Gene set difference analysis

Gene set variation analysis (GSVA) is a non-parametric, unsupervised method for assessing transcriptome gene set enrichment. GSVA converts gene-level changes into pathway-level changes by comprehensively scoring the gene sets of interest, thereby determining the changes in biological function. In this study, background gene sets will be downloaded from the MsigDB, and the GSVA algorithm will be used to comprehensively score each gene set to evaluate potential biological function changes across different groups.

#### 2.1.7 Transcriptional regulation analysis of key genes

This study utilized the R package “RcisTarget” to predict transcription factors, with all calculations performed by RcisTarget based on motifs. The normalized enrichment score (NES) of a motif depends on the total number of motifs in the database. In addition to the motifs annotated by the source data, we inferred additional annotation files based on motif similarity and gene sequence. The first step in estimating the overrepresentation of each motif in a gene set is to calculate the area under the curve (AUC) for each motif-gene set pair. This calculation is based on recovery curves of the gene set against the ordered motifs. The NES for each motif is then calculated based on the AUC distribution of all motifs in the gene set.

#### 2.1.8 Single-cell analysis

First, the expression profiles were read using the Seurat package, and low-expression genes were filtered out. The data were then standardized, normalized, and subjected to PCA analysis sequentially. The positional relationship between each cluster was determined through t-SNE analysis. Each cluster was subsequently annotated and analyzed separately. Important cells related to the occurrence of diseases were identified and annotated. Finally, marker genes for each cell subtype were extracted from the single-cell expression profiles using the FindMarkers function.

#### 2.1.9 Candidate drug prediction and molecular docking

Assessing protein-drug interactions is crucial for determining whether target genes can serve as viable drug targets. In this study, we used the Drug-Gene Interaction Database (DGIdb 4.0, https://www.dgidb.org/) for this purpose. DGIdb is a comprehensive database containing over 50,000 drug-gene interactions involving 10,000 genes and 15,000 drugs. The identified target genes were uploaded to DGIdb, allowing for the prediction of drug candidates to evaluate the medicinal potential of these target genes.

To further understand the effect of drug candidates on target genes and the druggability of these genes, this study also performed molecular docking at the atomic level to evaluate the binding energy and interaction patterns between drug candidates and their targets. Molecular docking simulations enable us to analyze the binding affinity and interaction modes between ligands and drug targets. By identifying ligands with high binding affinity and favorable interaction patterns, we can prioritize drug targets for further experimental validation and optimize the design of potential drug candidates. In this study, Autodock Vina v.1.5.6, a computerized protein-ligand docking software, was used to perform molecular docking of the most significant drugs and the proteins encoded by the corresponding target genes. Drug structure data were obtained from DrugBank Online^5^, and the corresponding IDs are listed in Table 1. Protein structure data were downloaded from the Protein Data Bank (PDB, http://www.rcsb.org/), with the corresponding PDB IDs also shown in Table 1. Final structures were obtained for three proteins and three drugs. We removed all water molecules from the protein and ligand files and added polar hydrogen atoms. The grid boxes were centered to cover the structural domains of each protein, allowing unrestricted movement of the molecules. The grid points of the docking pockets were spaced at 0.04 nm. The entire molecular docking process was visualized using Autodock Vina v.1.5.6.

**Table 1:** Docking results of available proteins with small molecules.

Target	PDB ID	Drug	DrugBank ID	Bingding energy
PPP1CB	1S70	MITOGLITAZONE	DB11721	−6.58
CSNK1E	4HNI	UMBRALISIB TOSYLATE	DB14989	−7.70
BHLHE40	Q6NY50 (UNIPROT)	TRICHOSTATIN A	DB04297	−5.86

*The lower the binding energy, the better the binding effect and the higher the affinity.

### 2.2 Statistical analysis

All data processing, statistical analysis, and plotting were conducted in R 4.2.0 software. Correlations between two continuous variables were assessed via Pearson’s correlation coefficients. Continuous variables were analyzed by Wilcoxon rank-sum test or Student’s *t*-test. Categorical variables were statistically compared using Chi-square test or Fisher’s exact test. The C-indices of different variables were compared using the CompareC package. The ROC used to predict binary categorical variables was implemented via the pROC package. The time-dependent area under the ROC curve (AUC) for survival variables was conducted by the timeROC package.

We set a Type I error acceptance threshold of 0.05, whereby original *p*-values obtained from the IVW method underwent FDR correction. Corrected *p*-values <0.05 were deemed indicative of significant causal relationships. Results with an uncorrected *p*-value <0.05 but a corrected *p*-value >0.05 were interpreted as potentially suggestive of underlying causal relationships, with causal effect estimates presented as OR and accompanied by 95% CI. All statistical tests were two-sided. *p* < 0.05 was regarded as statistically significant.

## 3 Results

### 3.1 Selection of characteristic genes via machine learning

**Figure 2:**
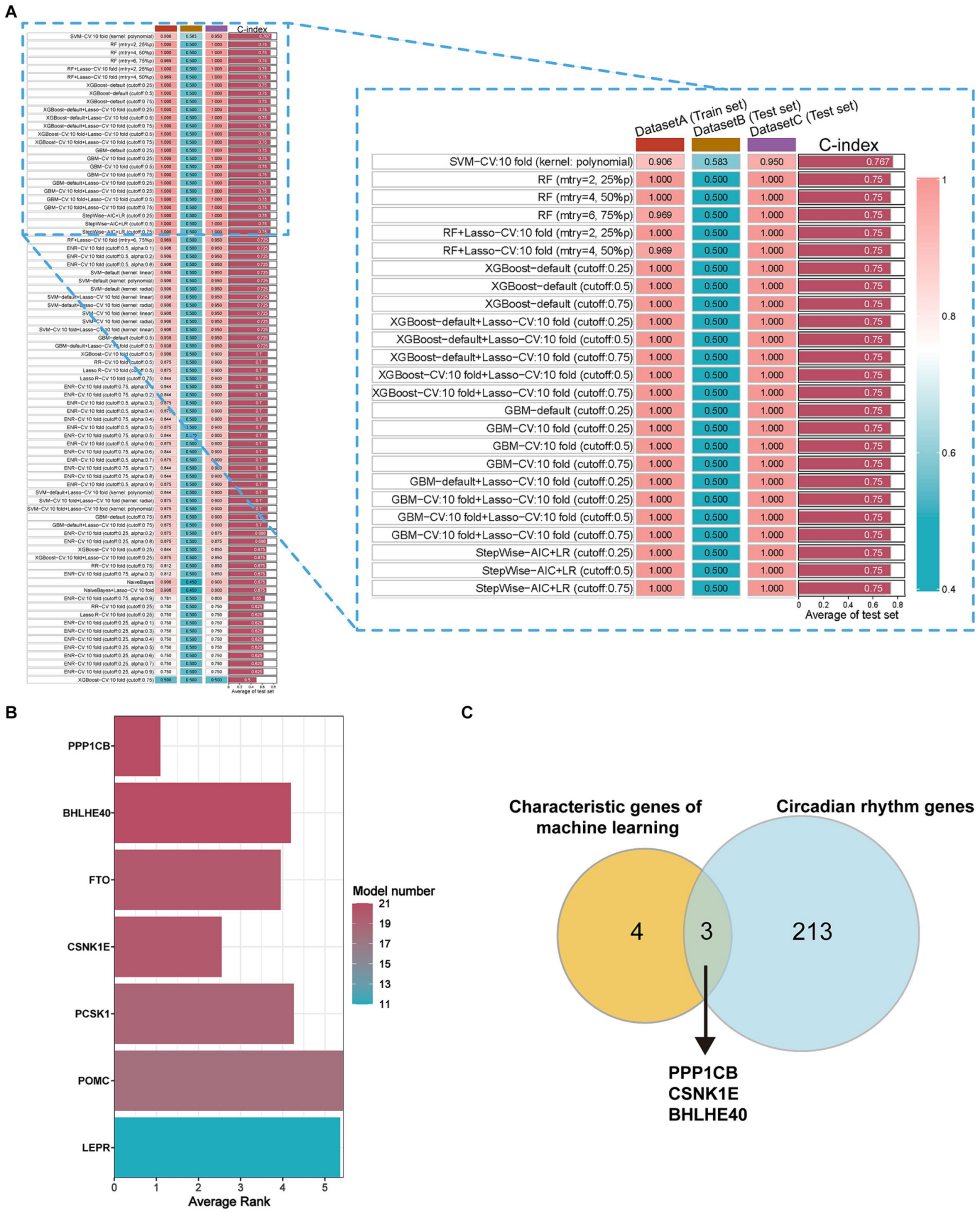
Selection of characteristic genes among DEGs in obesity via 80 machine-learning algorithm combinations. **(A)** C-index of estimating diagnostic efficacy in the multiple combinations of algorithms. DatasetA (Train set): GSE69039, DatasetB (Test set): GSE55008, DatasetC (Test set): GSE198012. **(B)** The average ranking represents for diagnostic performance of characteristic genes in different models. Model number: the numbers of 80 machine-learning algorithm combinations. **(C)** The key circadian genes between characteristic genes of machine learning and circadian rhythm genes. Characteristic genes: genes with model number >10 and average rank <6, DEGs, different expression genes.

80 machine-learning algorithm combinations were applied to select characteristic genes among DEGs in obesity. The SVM-CV (kernel: polynomial) with 10-fold cross-validation yielded the best C-index (Figure 2A). Seven characteristic genes were identified: PPP1CB, BHLHE40, FTO, CSNK1E, PCSK1, POMC, and LEPR (Figure 2B). To evaluate the robustness, we also calculated the *F*-score and accuracy for each model. Additionally, we used external independent datasets (GSE241015, GSE231656, and GSE133666) to verify the predictive performance, which demonstrated good results (Supplementary Table S1).

### 3.2 Mendelian randomization analysis

**Figure 3:**
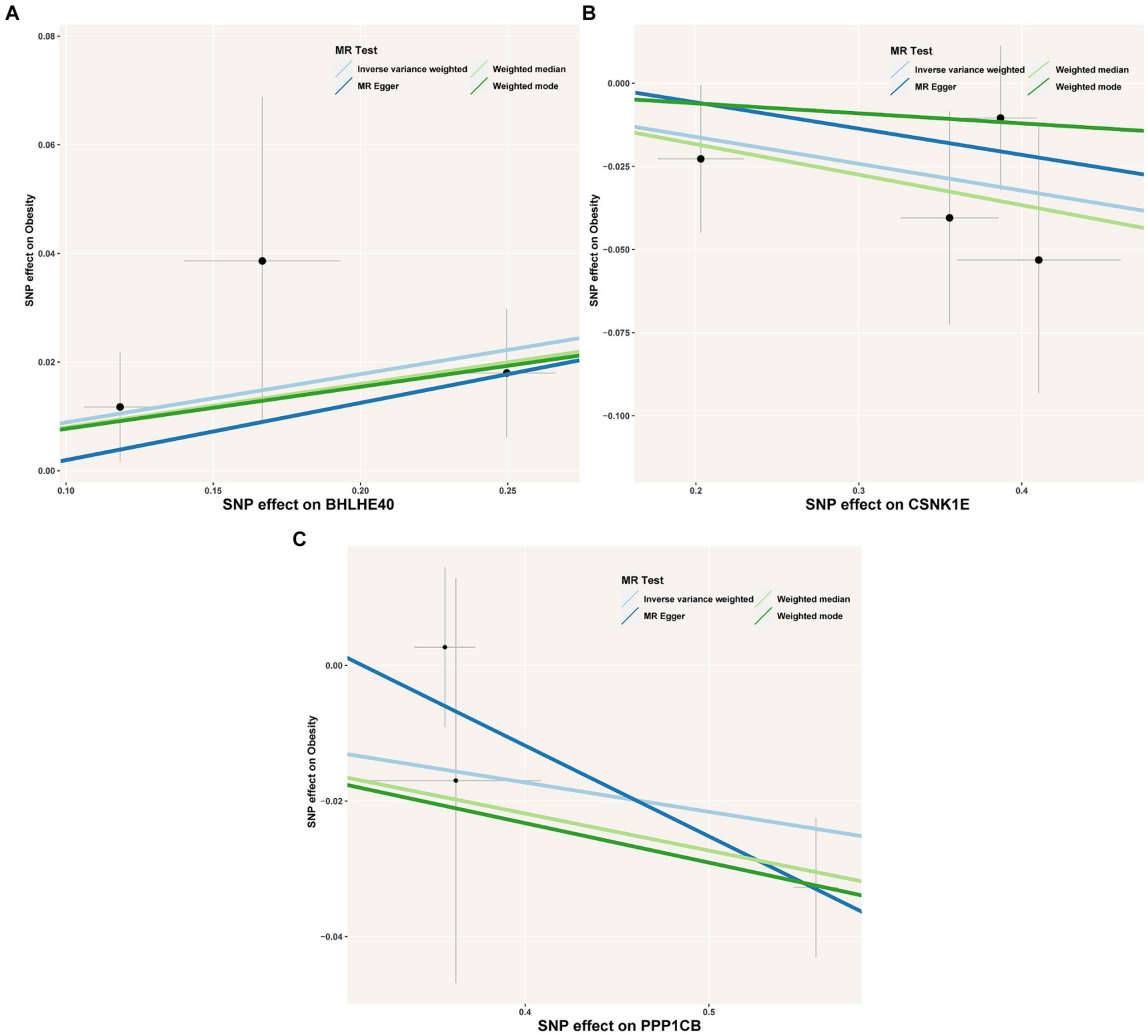
**(A–C)** Scatter plot showing the association of the SNP effects on obesity against the SNP effects on the expression of BHLHE40. The lines indicate the estimate of the effect using the inverse-variance weighted, MR-Egger, weighted mode, and weighted median method. Circles indicate marginal genetic associations with obesity and risk of BHLHE40, CSNK1E, and PPP1CB expression for each variant. Error bars indicate 95% CIs. SNP, single nucleotide polymorphism; eQTL, expression quantitative trait loci.

**Figure 4:**
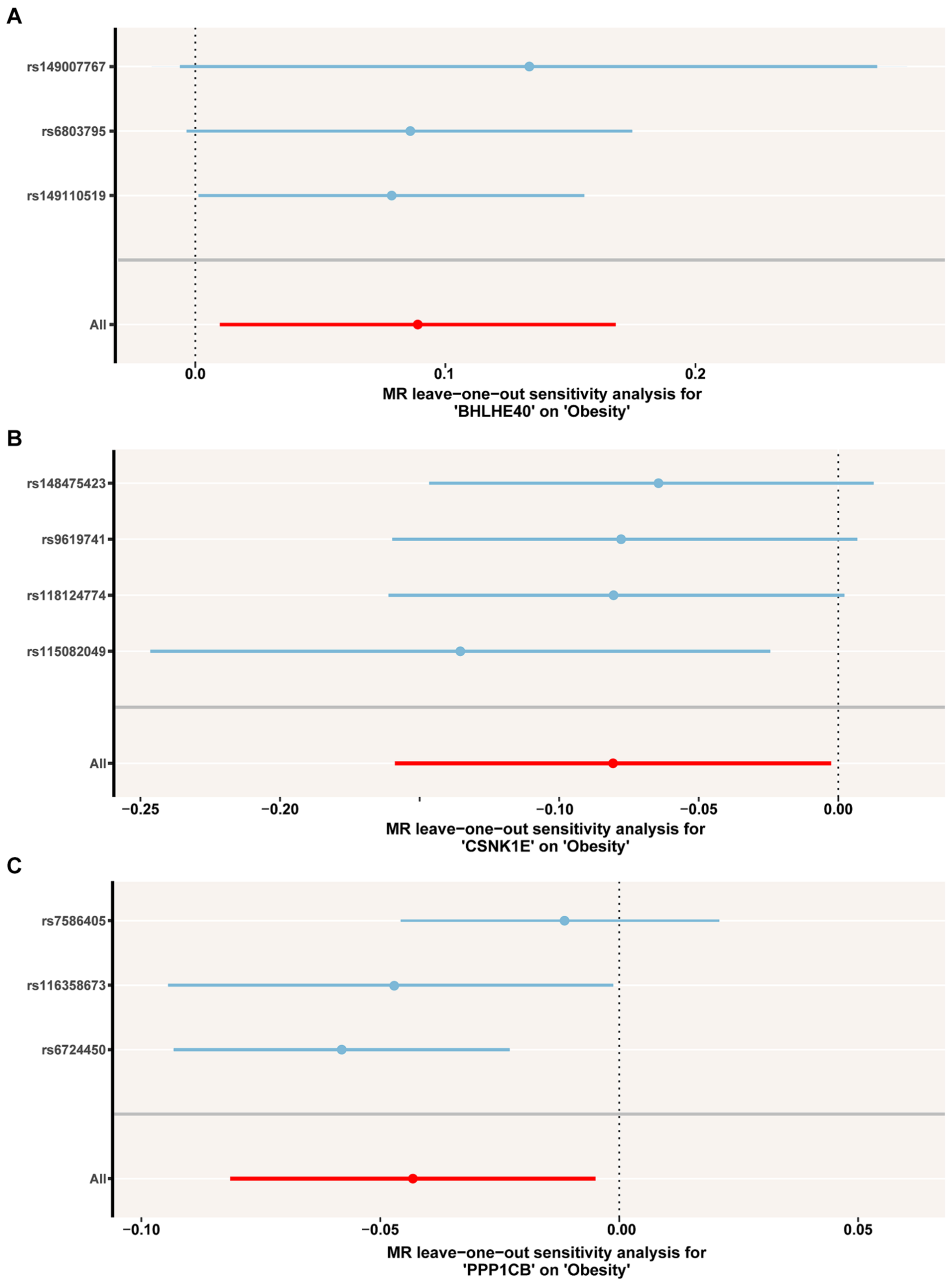
**(A–C)** Leave-one-out permutation analysis of the causal association between BHLHE40, CSNK1E, PPP1CB and obesity. MR, Mendelian randomization; SNP, single nucleotide polymorphism.

To identify the circadian-related genes in obesity, we downloaded circadian-related genes from the Genecard database^6^ and extracted 216 circadian rhythm genes (Supplementary Table S2) with relevance scores greater than 5. Three key genes identified through machine learning overlapped with the circadian genes (Figure 2C). Using the “extract instruments” and “extract outcome data” functions, we determined the causal correlations of three pairs of genes corresponding to the positive eQTL outcomes (Figure 3). The corresponding genes are BHLHE40, CSNK1E, and PPP1CB. The expressions of CSNK1E (OR: 0.922, 95% CI: 0.853–0.997, *p* = 0.043) and PPP1CB (OR: 0.958; 95% CI: 0.922–0.995; *p* = 0.027) may be associated with a lower risk of obesity, while BHLHE40 (OR: 1.093, 95% CI: 1.010–1.183, *p* = 0.0028) may be associated with a higher risk of obesity (Supplementary Table S3). Sensitivity analysis and reverse causality were performed on the causal relationships of the three genes to determine their reliability (Supplementary Figure S4). The results show that excluding any SNP has no significant impact on the overall error bar, indicating that the selected causal relationships are robust (Figure 4). Therefore, these three genes are key candidates for our subsequent investigation.

### 3.3 Immune cell infiltration analysis

**Figure 5:**
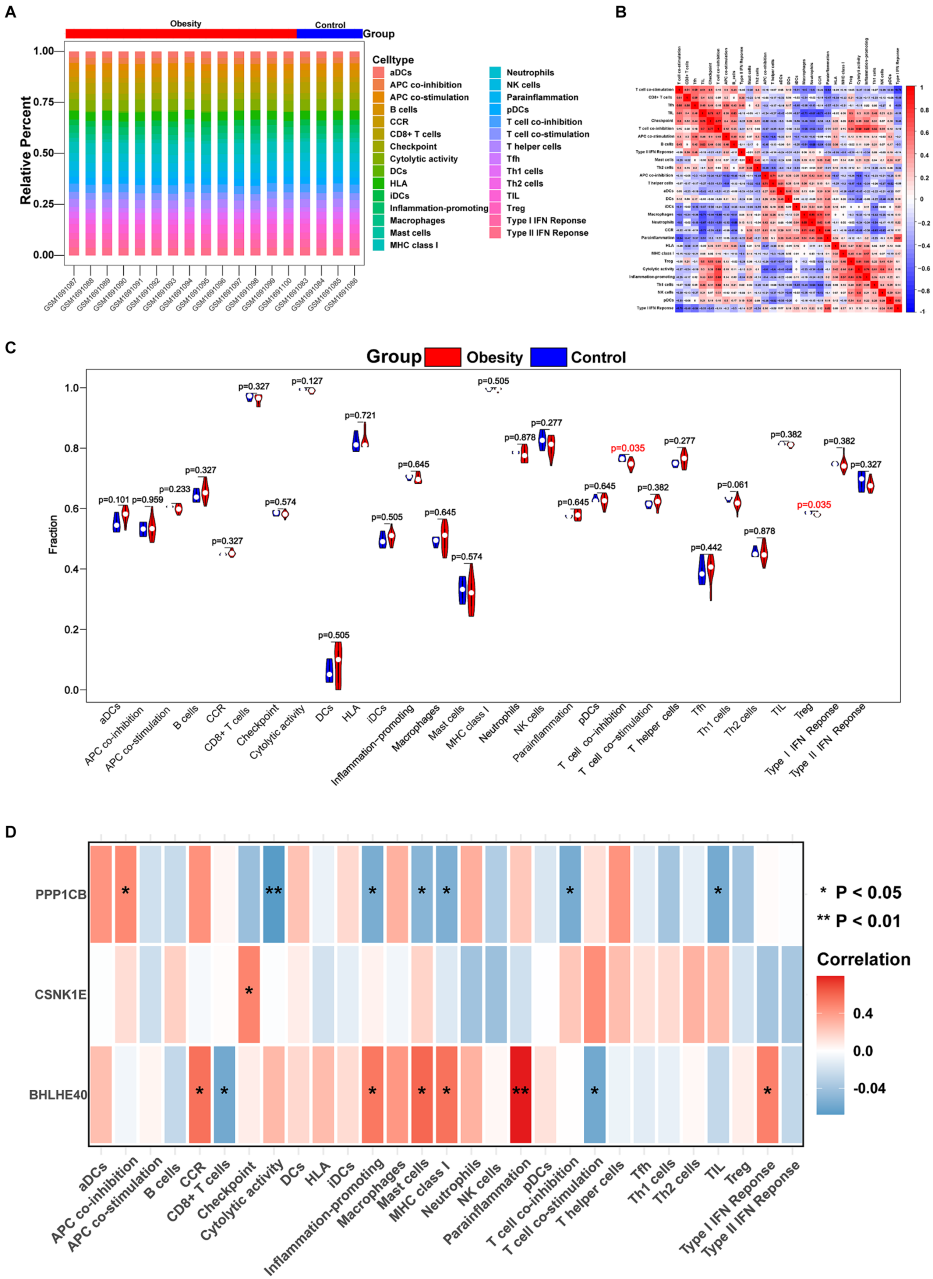
Immune cell infiltration in obesity and normal patients. **(A)** Histogram showing the composition of 29 immune cell phenotypes in each sample. **(B)** The correlations of 29 immune cell phenotypes in obesity. **(C)** Identifying the significantly different immune cells in obesity and normal patients by Wilcoxon test. **(D)** The correlations of 29 immune cell phenotypes and BHLHE40, CSNK1E, PPP1CB. Red: positive correlation; Blue: negative correlation. *p < 0.05 and **p < 0.01.

**Figure 6:**
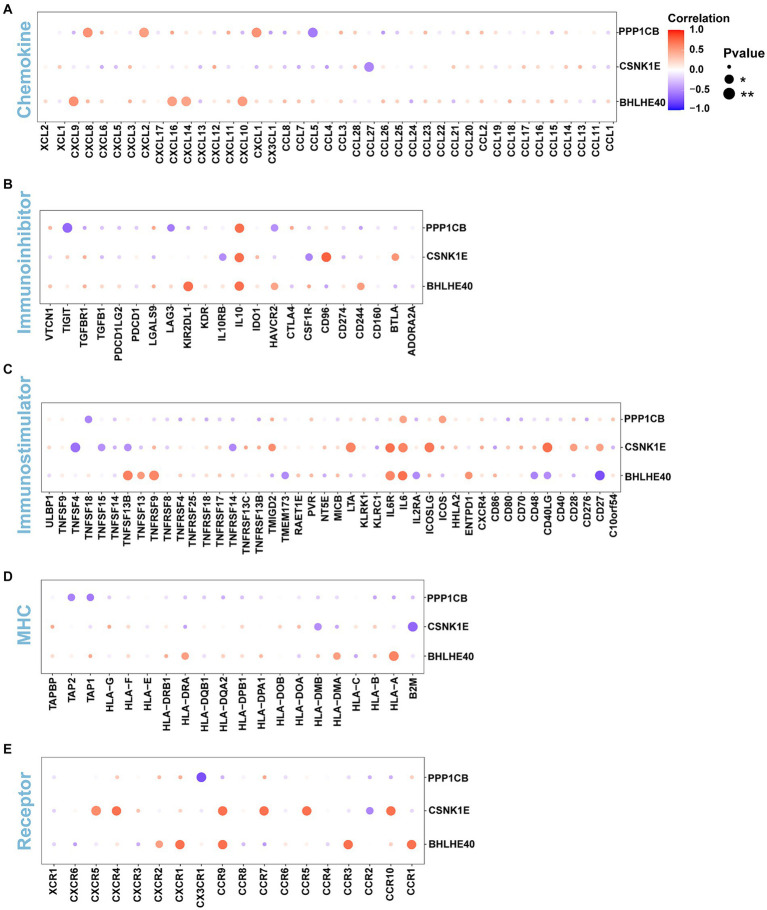
**(A–E)** The correlations of BHLHE40, CSNK1E, PPP1CB and chemokine, immunoinhibitor, immunostimulator, MHC, immunoreceptor. MHC, major histocompatibility complex.

The immune microenvironment is composed of immune cells, extracellular matrix, various growth factors, inflammatory factors, and unique physical and chemical characteristics. It significantly influences disease diagnosis and the sensitivity of clinical treatments. This study demonstrates the proportion of immune cells in each sample and the correlations between different immune cells (Figures 5A,B). The results indicate that T cell co-inhibition and Treg cells show statistically significant differences in expression between groups (Figure 5C). Further analysis revealed that BHLHE40 is significantly positively correlated with chemokine receptor (CCR), para-inflammation, and inflammation-promoting, and significantly negatively correlated with CD8^+^ T cells and T cell co-stimulation. CSNK1E is significantly positively correlated with checkpoint markers, while PPP1CB shows a significant positive correlation with APC co-inhibition and a significant negative correlation with cytolytic activity, inflammation-promoting, and mast cells (Figure 5D). Additionally, we obtained correlations between key genes and various immune factors, including immunosuppressive factors, immunostimulatory factors, chemokines, and receptors, from the tumor-immune system interactions database (TISIDB, http://cis.hku.hk/TISIDB/) (Figure 6). These analyses suggest that key genes are closely related to the level of immune cell infiltration and play a crucial role in the immune microenvironment.

### 3.4 GSEA pathway enrichment analysis

**Figure 7:**
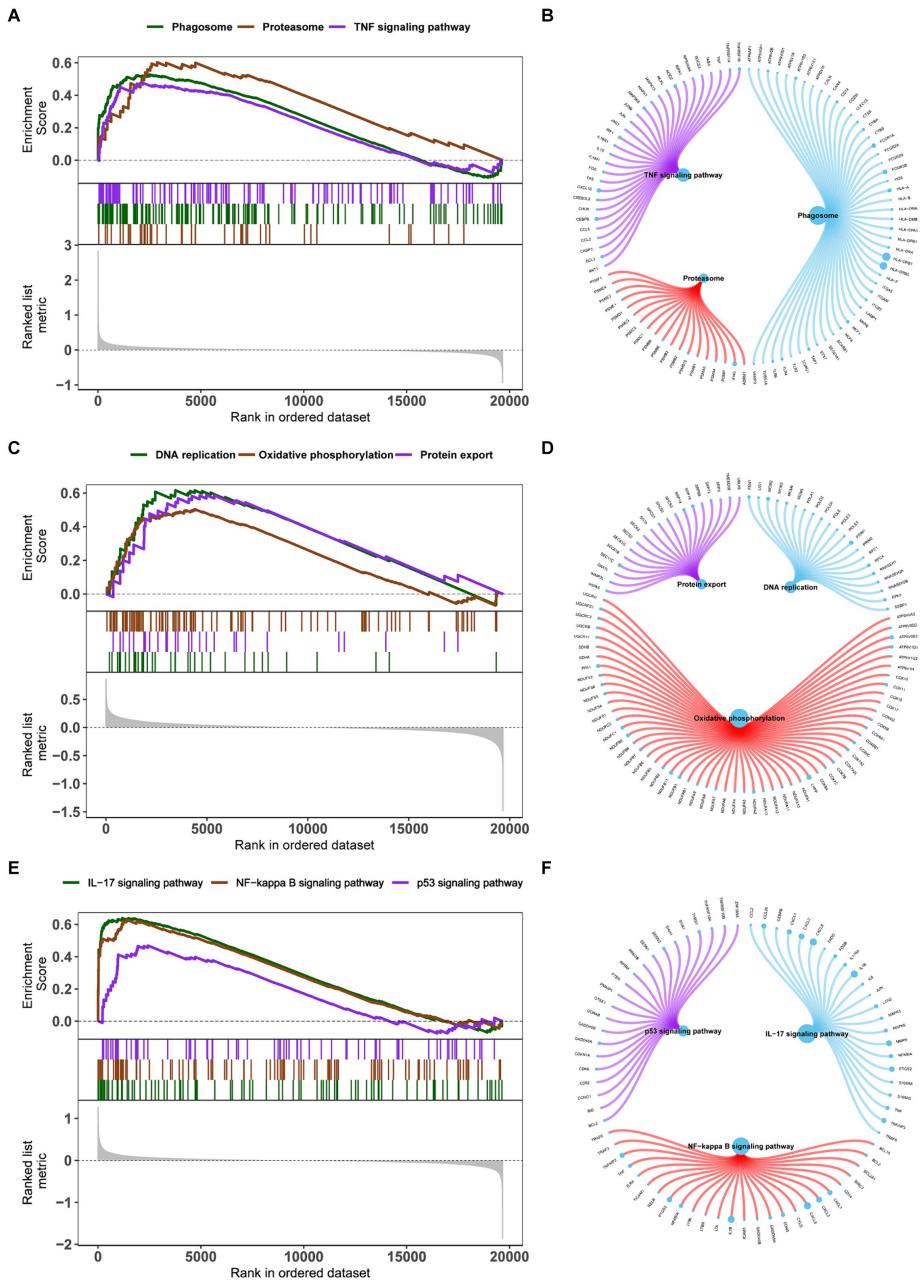
**(A,C,E)** Enrichment plots for the representative pathways in obesity by GSEA function analysis. **(B,D,F)** Genes in the representative pathways by GSEA function analysis. GSEA, gene set enrichment analysis.

The specific signaling pathways are enriched in three key genes to explore the potential molecular mechanisms by which key genes affect the progression of obesity. The GSEA results show that the pathways enriched by BHLHE40 include the Phagosome, Proteasome, and TNF signaling pathways (Figures 7A,B). Pathways enriched by CSNK1E include DNA replication, Oxidative phosphorylation, and Protein export (Figures 7C,D). Pathways related to PPP1CB include the IL-17, NF-κB, and P53 signaling pathways (Figures 7E,F).

### 3.5 GSVA pathway enrichment analysis

**Figure 8:**
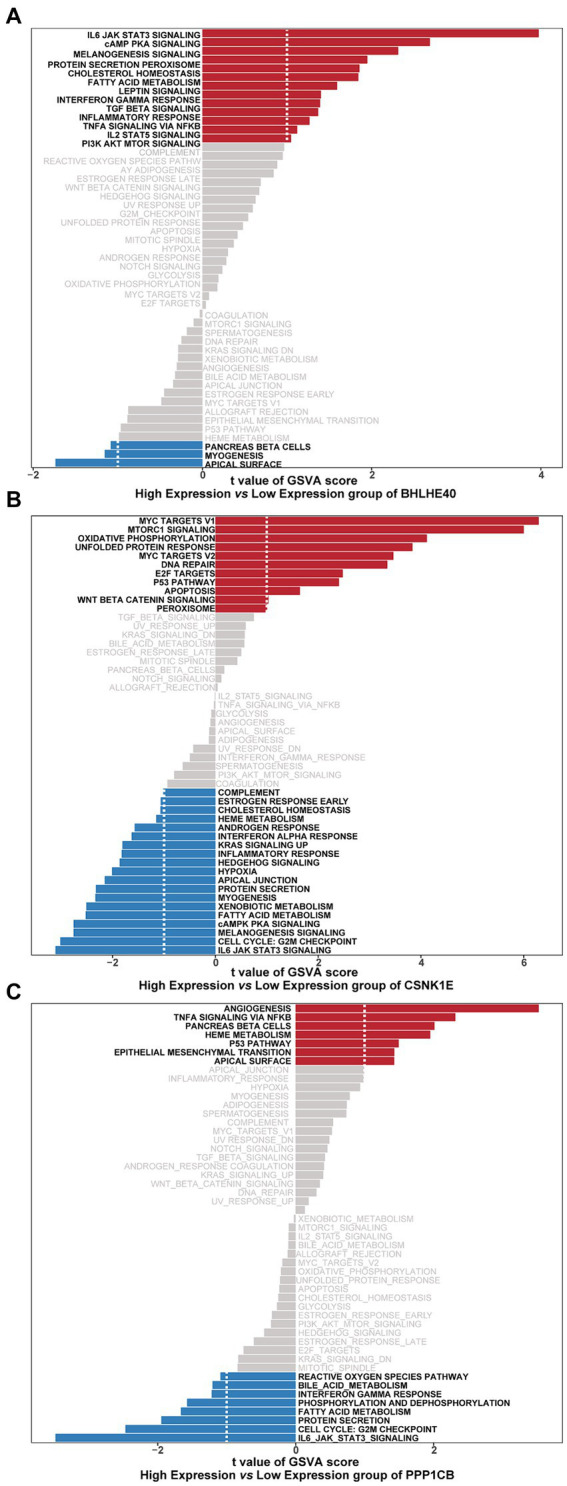
Gene sets enriched in samples with the expression of BHLHE40 **(A)**, CSNK1E **(B)**, or PPP1CB **(C)** by GSVA function analysis. Red: High expression; Blue: Low expression. GSVA, gene set variation analysis.

GSVA results show that high-expression BHLHE40 is enriched in IL6 JAK STAT3, cAMP PKA, and fatty acid metabolism pathways (Figure 8A). CSNK1E is mainly enriched in signaling pathways such as IL6 JAK STAT3, G2M Checkpoint, and P53 pathways (Figure 8B). PPP1CB is enriched in TNFα signaling, IL6 JAK STAT3, and G2M Checkpoint, and P53 pathways (Figure 8C). This suggests that key genes may influence disease progression through these pathways.

### 3.6 The relationship between obesity regulatory genes and key genes

**Figure 9:**
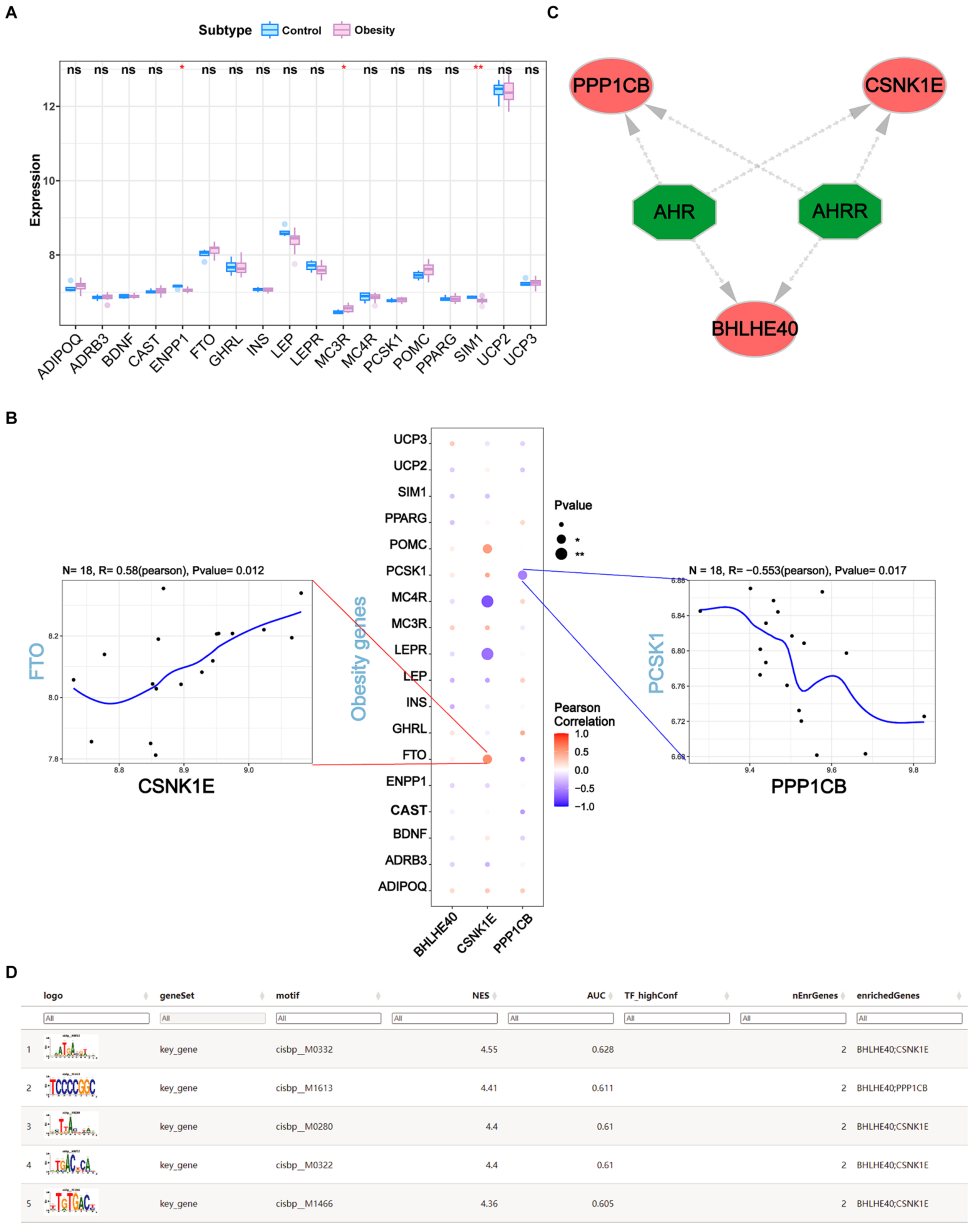
**(A)** The expression levels of the top 18 obesity-related genes coding protein in obesity and normal patients. **(B)** The correlations of BHLHE40, CSNK1E, PPP1CB and top 18 obesity-related genes by Pearson analysis. **(C,D)** Transcriptional regulation analysis of key genes using RcisTarget. ns, no significance (Student’s *t*-test). *p < 0.05 and **p < 0.01.

Obesity-related genes were obtained through the GeneCards database. We analyzed the expression levels of obesity-related genes with the top 20 relevance score (Supplementary Figure S5) and analyzed the expression of genes coding protein between groups. It was found that the expressions of ENPP1, MC3R, and SIM1 were significantly different between controls and obesity (Figure 9A). Pearson correlation analysis was performed and we found that the expression levels of key genes were significantly correlated with the obesity-related genes. Among them, CSNK1E was significantly positively correlated with FTO (*R* = 0.58, *p* < 0.05), and PPP1CB was negatively correlated with PCSK1 (*R* = −0.553, *p* < 0.05) (Figure 9B).

### 3.7 Transcriptional regulation analysis of key genes

We used three key genes (BHLHE40, CSNK1E, and PPP1CB) for this analysis and found that they are regulated by common mechanisms, including multiple transcription factors. Consequently, we performed an enrichment analysis of these transcription factors using cumulative recovery curves. Motif-TF annotation and analysis results of important genes revealed that the best motif—cisbp_M0332 and its highest NES is 4.55. We displayed all enriched motifs and corresponding transcription factors of key genes using Cytoscape (Figures 9C,D).

### 3.8 Single-cell sequencing analysis

**Figure 10:**
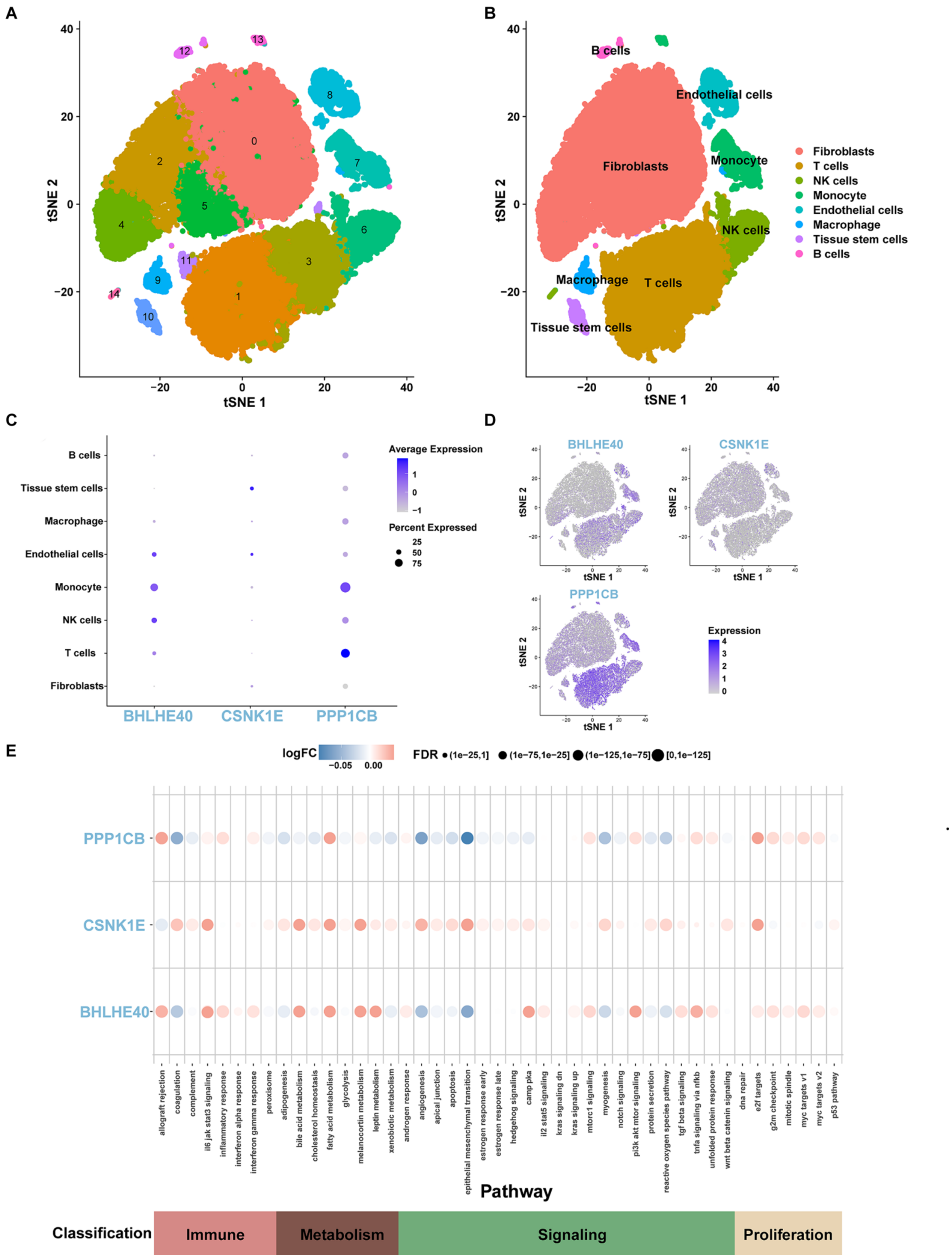
**(A)** tSNE representation of human adipose cells colored into 15 distinct clusters. **(B)** tSNE plot of human adipose cells colored by 8 major cell types in the obesity group by SingleR algorithm. **(C,D)** The expression levels and distribution of BHLHE40, CSNK1E, and PPP1CB in 8 types of cells. **(E)** The expression levels of BHLHE40, CSNK1E, and PPP1CB in immune, metabolism, signaling, and proliferation pathways on the single-cell data using the AUCell function.

We conducted the single-cell analysis using the Seurat package. The clustering of the cells performed by the tSNE algorithm results in 15 subtypes (Figure 10A). Each subtype was annotated, and the 15 clusters are categorized into 8 cell types: fibroblasts, T cells, NK cells, monocytes, endothelial cells, macrophages, tissue stem cells, and B cells (Figure 10B). The expression patterns of key genes across 8 cell types are presented in Figure 10C,D. Furthermore, co-expression analysis of obesity-regulated genes and key genes in single-cell data reveals the correlations among them (Supplementary Figures S6, S7). The result of AUCell analysis shows immune and metabolic pathways in different cell types and illustrates the associations between key genes and immune, metabolic pathways (Figure 10E).

### 3.9 Candidate drug prediction and molecular docking

**Figure 11:**
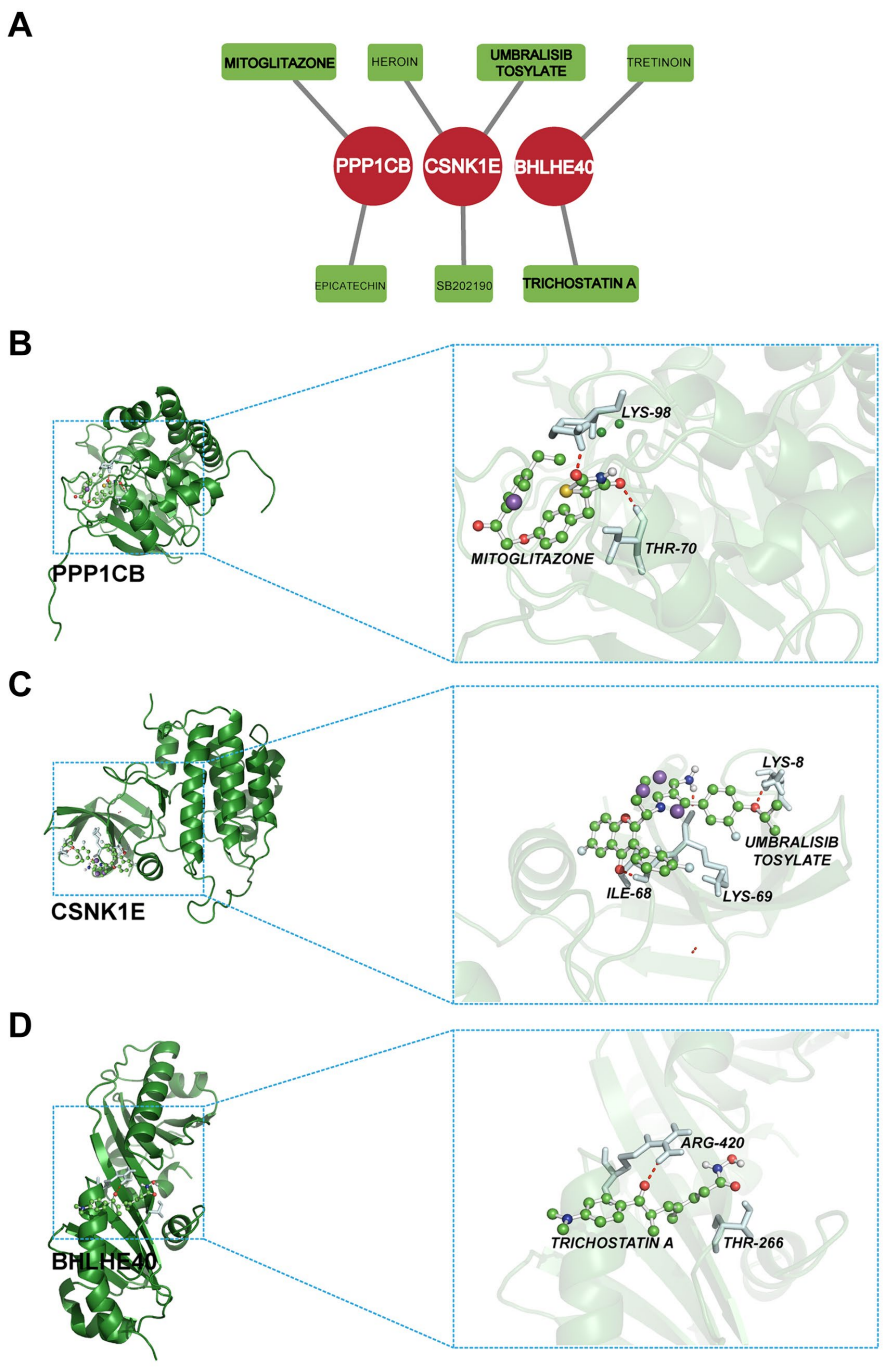
**(A)** Candidate drug predicted using DGIdb. **(B–D)** Docking results of available proteins and small molecules. **(B)** PPP1CB docking Mitoglitazone, **(C)** CSNK1E docking Umbralisib tosylate, **(D)** BHLHE40 docking Trichostatin A.

In this study, the potential compounds were displayed based on interaction scores (Figure 11A). Our findings revealed that Mitoglitazone and Umbralisib tosylate emerged as the most significant drugs associated with PPP1CB and CSNK1E. Additionally, Trichostatin A exhibited the strongest interaction with BHLHE40. To assess the drug candidates’ affinity for the target and understand the druggability of the drug target, this study conducted molecular docking experiments. The results yielded three proteins with validated docking outcomes for the drugs (Table 1 and Figures 11B–D). Each drug candidate formed visible hydrogen bonds and strong electrostatic interactions with its protein target. Furthermore, each target’s binding pocket was effectively occupied by drug candidates. Mitoglitazone, Umbralisib tosylate, and Trichostatin A demonstrated low binding energies (−6.58, −7.70, −5.86 kcal/mol), indicating highly stable binding.

### 3.10 Key genes in chronic obese mice induced by high-fat diet

**Figure 12:**
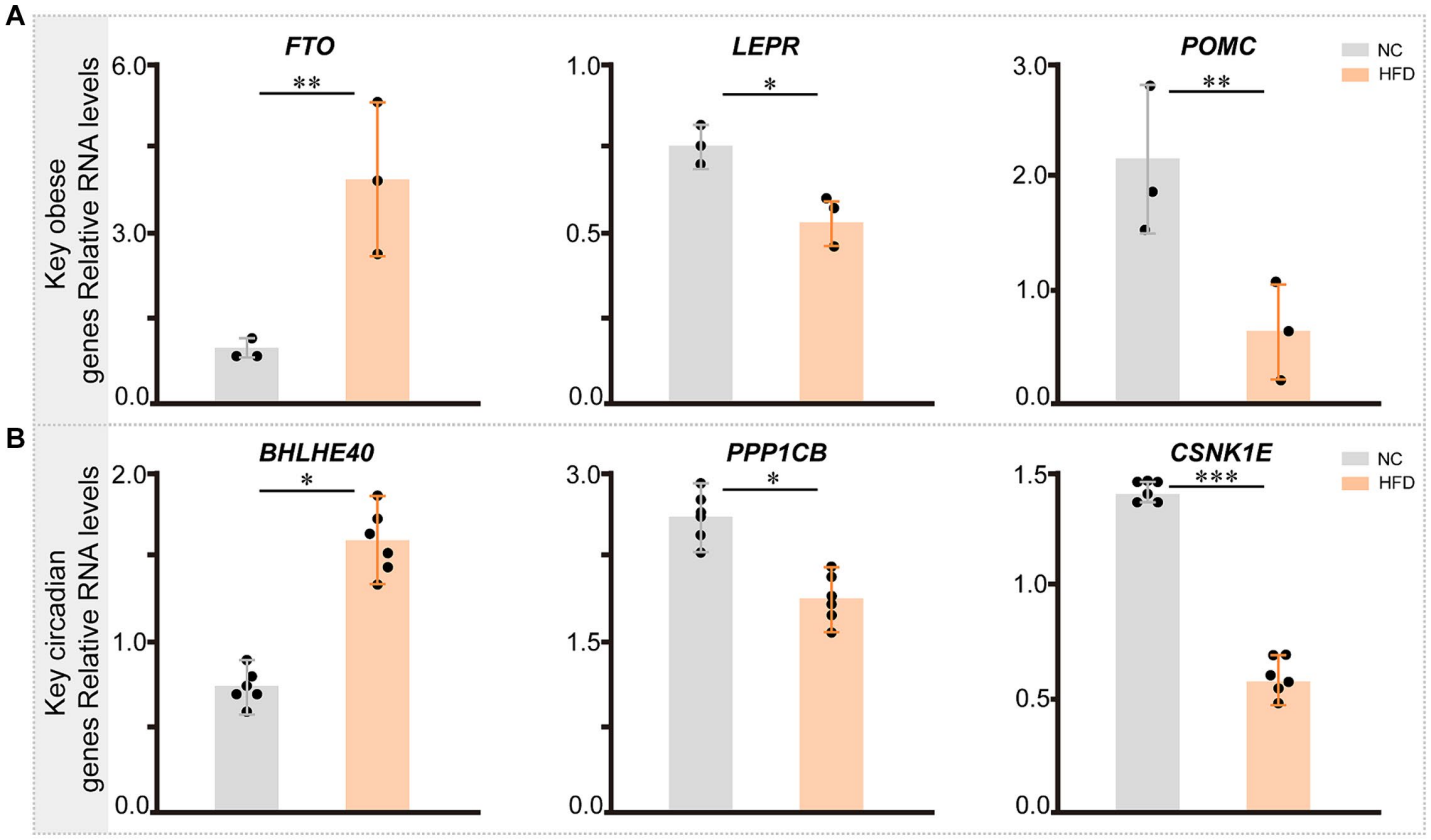
The expression of key genes related with circadian rhythm and obesity in NC- and HFD-fed mice. **(A)** The expression pattern of key genes related with obesity across different groups in mice (*n* = 3). **(B)** The expression pattern of key circadian genes across different groups in mice (*n* = 6). Statistical analysis was performed using Student’s *t* test. Data are expressed as mean ± standard deviation. *p < 0.05, **p < 0.01, and ***p < 0.001.

We validated the reliability of the model by assessing mouse body weight, serum glucose levels, glucose tolerance test, blood biochemistry test (total lipid, low-density lipoprotein cholesterol, high-density lipoprotein cholesterol, and triglyceride), and classical obesity-related genes (Supplementary Figure S8). Subsequently, we collected epididymal, mesenteric, perirenal, and retroperitoneal adipose tissues for quantitative polymerase chain reaction (qPCR), revealing that the expression levels of SERPINE1 (*p* < 0.05) and FTO (*p* < 0.01) were significantly elevated in the HFD group, while LEPR (*p* < 0.05), POMC (*p* < 0.01), PPP1CB (*p* < 0.05), and CSNK1E (*p* < 0.001) were downregulated (Figures 12A,B).

## 4 Discussion

**Figure 13:**
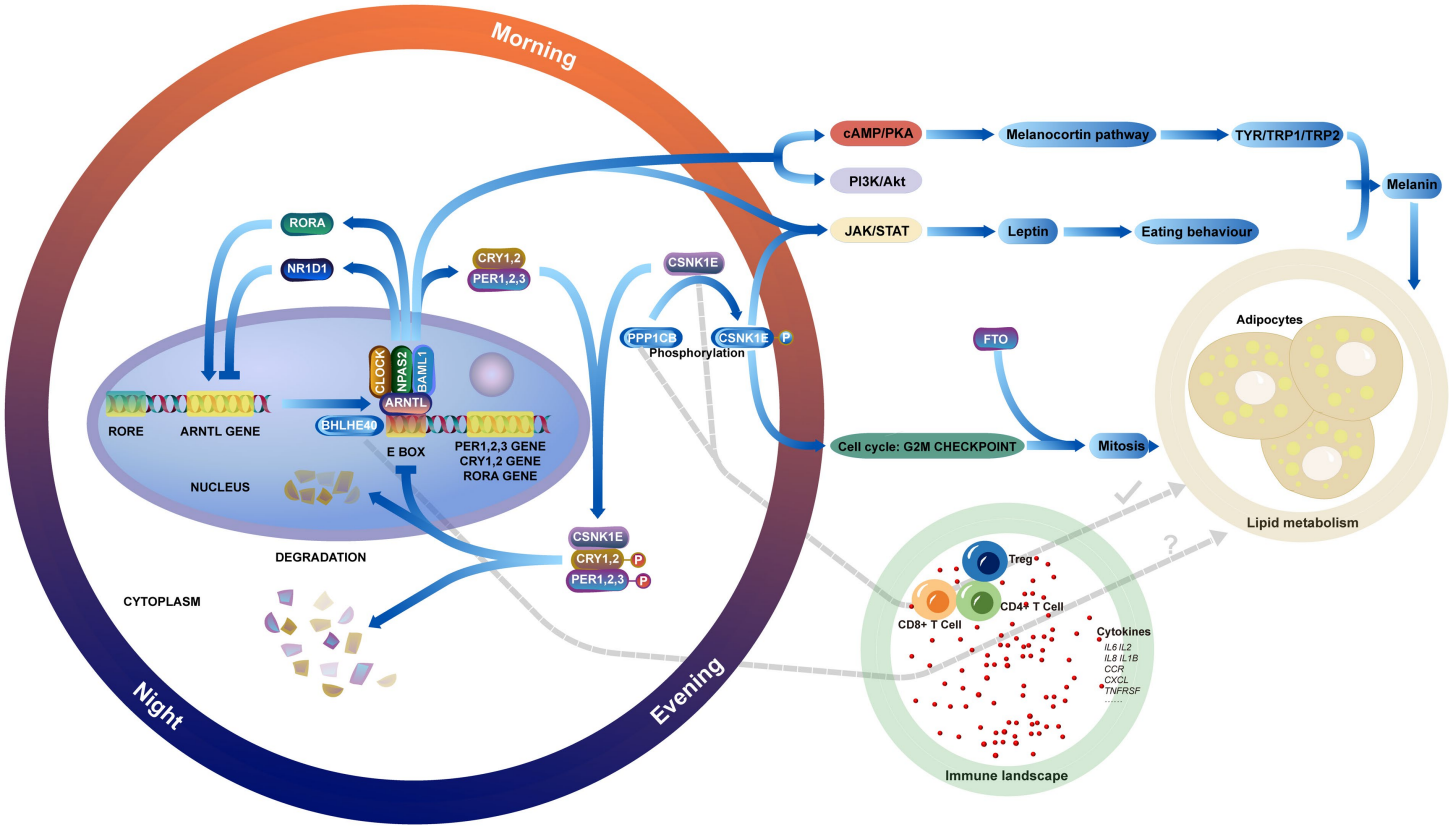
Showing the relationship between circadian genes, lipid metabolic pathways in obesity, the immune landscape, and the interactions between them.

Obesity, a complex disease influenced by numerous factors such as socioeconomic status, lifestyle, genetics, environmental conditions, and psychological health, has become a global health concern affecting individuals of all ages and regions. The incidence of obesity is steadily increasing especially in developed countries. The transformation of modern lifestyles, mental disorders, and the interaction of genetic and environmental factors play critical roles in the occurrence and development of obesity (26). Moreover, obesity and its complications, such as diabetes and cardiovascular diseases, increase the risk of mortality (27). Current management strategies for obesity include lifestyle modifications (28), anti-obesity drugs (29, 74–78), and bariatric surgery (30, 79–81). Due to the absence of specific pharmacological interventions, lifestyle changes remain crucial in managing obesity (31) and reduce the risk of cardiovascular diseases (32). However, irregular lifestyles exacerbate the incidence of obesity. Mutations in circadian genes are linked to diet-related obesity, and high-fat diets can disrupt normal circadian rhythm and sleep. This suggests a bidirectional regulatory relationship between the biological clock and metabolism (33). Despite extensive studies on the role of circadian rhythm in lipid metabolism at the levels of gene and protein in rodents (34), the specific regulatory mechanisms remain largely unknown. Therefore, it is essential to delve deeper into the pathogenesis of obesity to uncover the molecular regulatory mechanisms of key genes involved in obesity development (Figure 13). This could shed light on new therapeutic strategies and the development of targeted medicines for obesity.

Previously, there was limited research utilizing MR analysis to explore the relationship between circadian genes and obesity. In our study, associations were established between three circadian genes and obesity through MR analysis. However, an experimental study found that BHLHE40 is closely related to lipid metabolism, with gene knockdown significantly reducing lipid levels and oxidative stress (35). Additionally, BHLHE40, as an essential transcription factor of sterol-responsive element-binding protein (SREBP), regulates fat synthesis (36). These studies confirm that high levels of BHLHE40 expression might increase the risk of obesity. Previous observational studies have shown no significant association between the genetic variation of CSNK1E and body mass index (BMI) (37). However, our forward and reverse MR analyses indicate a significant association with obesity and a reduced risk of obesity. We believe this discrepancy may be influenced by potential confounding factors, biases in reverse causality, and sample size. Regarding PPP1CB, it has been shown to impact adipogenesis in mice by regulating the early adipogenesis process (38). However, there have been no reports on the association between PPP1CB expression levels and obesity in humans. Our MR results found a significant causal relationship between PPP1CB and obesity, suggesting that inhibition of PPP1CB may reduce the risk of obesity. Although the biological rationale for the association between disrupted circadian rhythms and obesity risk is sound, more evidence is needed to elucidate their mechanisms. This includes preventive randomized controlled trials and animal experiments to provide insights into molecular biology.

The key circadian genes play crucial regulatory roles in lipid metabolism. BHLHE40, a transcriptional suppressor, competes for the E-box binding site in the promoter of PER1 and inhibits the activation of CLOCK-BMAL1/BMAL2 heterodimers (39, 82), which activates TAK/STAT pathway and increases the plasma concentration of leptin. Patients develop leptin resistance, disrupt neuropeptide synthesis, and impair vagus nerve input neuron signaling. This disruption leads to altered food intake behavior, energy consumption, and lipid metabolism, resulting in type A obesity (6). Furthermore, the negative regulation of circadian genes by BHLHE40 gradually leads to circadian disruption in central and peripheral organs, potentially affecting lifestyle choices and exacerbating obesity. BHLHE40 may also contribute to the regulation of chondrocyte differentiation via the cAMP pathway (40). However, research on the implications of BHLHE40 in the pathogenesis of obesity is relatively limited, with most studies focusing on the immune response (41, 83, 84). Additionally, BHLHE40 contributes to expression of TYR/TRP1/TRP2 and melanogenesis by activating the cAMP-regulated melanocortin signaling and PI3K/Akt pathways through protein secretion, leading to an imbalance in energy homeostasis.

CSNK1E, a serine/threonine protein kinase, determines the length of the circadian rhythm by controlling the nuclear transport, phosphorylation, and degradation of PER1 and PER2 (42, 85). PPP1CB, also a serine/threonine protein kinase, can regulate circadian rhythm by modulating the phosphorylation and dephosphorylation of CSNK1E. Abnormal expressions of PPP1CB and CSNK1E in obesity directly affect the core clock genes (PER1, PER2), and the accumulation of PERs and CRYs in the nucleus inhibits the transcription of CLOCK and BMAL1, disrupting energy homeostasis and leading to obesity. Considering the correlation between CSNK1E and FTO, it is plausible that CSNK1E may disrupt normal fat metabolism and energy homeostasis by regulating FTO expression (43, 86, 87). These two genes regulate the mitosis of adipose precursor cells by affecting the cell cycle and increasing the prolipogenic factor RUNX1T1. Besides, the activation of the mTORC1 pathway promotes lipid *de novo* synthesis through SREBP transcription factors. Abnormal activation of this pathway stimulates the expression of metabolic genes associated with fatty acid and cholesterol biosynthesis.

Obesity is characteristically associated with chronic inflammation in adipose tissue, which intimately relates to the abnormal activation of the infiltration of inflammatory cells. Chronic inflammatory diseases observed in cases of severe obesity are characterized by persistent activation of the innate immune system (44, 88). Several immune molecules have been implicated in the pathogenesis of obesity, such as TNFα, which shows elevated plasma levels in obese patients (45). Additionally, studies report activated complement systems (46, 89) and acute phase proteins (c-reactive protein and α1-acid glycoprotein) (47) can be reduced after weight loss. The immune cells in obesity significantly contribute to the production of inflammatory mediators. Both clinical and experimental studies have demonstrated that adipose tissue macrophages (ATMs) secrete pro-inflammatory cytokines, including interleukin-1β (IL-1β) and IL-18, which are essential factors in the progression of obesity-related metabolic disorders (48). Our study revealed a significant correlation between key genes and acquired immunity, which differs from previous research findings. This indicates that circadian genes may influence the development of the obesity-related immune infiltration landscape through mechanisms other than regulating macrophages.

Studies have indicated that obesity modifies the immune status of the body. Researchers observed a notable increase in the count of CD4^+^ T cells, Treg cells, and CD8^+^ T cells in the obese mice induced by high-fat diets. This indicates an escalation in the overall inflammatory response (49). These findings align with the results of our study. Moreover, our research showed that BHLHE40 amplifies the inflammatory response in the pathogenesis of obesity by instigating the adaptive immune response of Treg cells and CD4^+^ T cells and promoting the chemotaxis of vital immune molecules, such as CCR, CXCL, and TNFRSF, in tissues. The pro-inflammatory property of BHLHE40 may also be related to its ability to enhance the expression of HIF1α in macrophages, thereby promoting the expression and function of inflammatory genes (50). Fujita et al. (35) found that knocking out BHLHE40 reduced lipid levels in mice and activated lysosomal activity in macrophages, which is involved in cholesterol clearance (51). Our study did not find expression differences of BHLHE40 in macrophages, suggesting that further single-cell studies based on immune cell populations may be needed. BHLHE40 expression is reduced in the livers of leptin-deficient and leptin receptor-deficient mice, with a significant increase in liver triglycerides (52). In contrast, mice with alcohol-induced fatty liver showed a slight increase in liver triglyceride as BHLHE40 expression increased (53). Additionally, DEC1 inhibits adipocyte differentiation by reducing the expression of peroxisome proliferator-activated receptor gamma (PPARγ) (54). These animal experiments differ from our study results, suggesting that other molecular changes regulated by BHLHE40 may contribute to the development of obesity.

Prior studies have reported that CSNK1E can regulate genes controlling T-cell division and promote the transcription and expression of cytokine genes by activating either the NF-κB1 or NF-κB2 pathway (55). In this study, CSNK1E, as an immune checkpoint, modulates the activation of the immune system by inhibiting the release of B2M and TNFSF4. Lower expression of CSNK1E could accelerate obesity-related inflammation. When circadian core genes (CSNK1E, NFKB2, and RORα) are highly expressed, they downregulate inflammatory response (IL1B and IL8) and lipid metabolism (ABCA1, ABCD1, and ABCG1) by NF-κB pathway (56), which aligns with our findings. However, our results indicate that CSNK1E primarily affects the immune status of obese patients, without direct evidence of its impact on lipid metabolism. PPP1 is a serine/threonine-specific protein phosphatase involved in regulating various cellular processes (57). The human catalytic subunit (PPP1C) is encoded by 3 separate genes (PPP1CA, PPP1CB, and PPP1CC). PPP1CB can dephosphorylate CSNK1E (58), suggesting that these two genes may contribute to the immune landscape in obesity collectively. Animal studies have indicated that PPP1CB regulates hepatic fatty acid synthesis by phosphorylating WDR6, disrupting triglyceride synthesis (59). PPP1CB might inhibit the progression of obesity by managing lipid metabolism directly. PPP1CB might exert pro-inflammatory effects by activating inflammatory pathways such as the IL-17 signaling pathway and NF-κB signaling pathway, which activate T cells and monocytes. However, no studies have focused on the role of PPP1CB in immune inflammation and pathogenesis in obesity. We postulate that obese patients may exhibit high expression of PPP1CB in immune cells, which might increase the dephosphorylation process of CSNK1E. This could potentially mitigate the anti-inflammatory effect of CSNK1E, promoting an increase in inflammatory factors and immune cell infiltration. This might represent a protective mechanism of the body against obesity. Additionally, obese patients often have obstructive sleep apnea (OSA) (60, 89, 90), and the expression levels of circadian genes have been shown to change in OSA (61, 91–93). This might provide a new potential mechanism linking obesity with OSA. Long-term sleep disorders in OSA patients disrupt the circadian rhythm, affecting normal metabolic pathways and leading to obesity. However, this is speculative, and the specific mechanism by which OSA leads to obesity is not yet clear.

The relationships between obesity and inflammation were first reported nearly 30 years ago (62), revealing that inflammatory factors secreted by infiltrating immune cells disrupt the function and metabolic dynamics of various metabolic cells, exacerbating the progression of obesity and metabolic diseases (63, 94–96). More recent studies have focused on the molecular changes in obesity-related inflammation, highlighting the roles of T cells and macrophages in promoting obesity (64, 97). These findings align with the results of our study. Furthermore, we analyzed the correlations between circadian genes immune cells, and cytokines, discovering that circadian genes are directly involved in the chronic inflammatory process of obesity. Evidence suggests that circadian disorders lead to systemic inflammation, supporting this view (65). Therefore, regulating the circadian rhythm can not only prevent and reduce obesity but also alleviate inflammatory responses in patients, restoring normal immune function and reducing the risk of complications such as metabolic disorders. To enhance the impact and applicability of our findings, we propose the following experiments. C57BL/6 J male mice aged 8–12 weeks will be used to create an obese model induced by high-fat diets, ensuring the avoidance of diabetes and other accompanying diseases. Daily recordings of blood sugar, body length, weight, and Lee’s index will be taken, with an index greater than 0.50 indicating obesity. Western blotting will be employed to measure the levels of gene-encoded proteins in the adipose tissue of obese and normal mice. We will also investigate characteristic genes and expression products of lipid metabolism pathways, as well as the NF-κB, IL-17, and P53 pathways. Correlations between key genes and pathways will be calculated to enhance our understanding of the molecular mechanisms involved. Additionally, we will explore the regulatory networks of genes using RNA pull-down, immunoprecipitation, and chromatin immunoprecipitation sequencing (ChIP-seq). These techniques will provide further insights into the interactions and regulatory mechanisms that govern gene expression in the context of obesity and inflammation.

So far, observational studies on the association between circadian disorders and obesity risk have been insufficient (6, 66, 98). Our study found significant associations between circadian genes and obesity, indicating that circadian disorders may not be conducive to preventing obesity. Therefore, healthcare providers should advise patients to maintain good sleep patterns and avoid unhealthy lifestyles, such as staying up late and shift work. Although meta-analyses have shown that circadian disorders increase lipid levels and promote obesity (67, 99), Keith et al. found no significant association between circadian disorders and BMI (68). Thus, the relationship between circadian rhythm and obesity remains controversial. We found that previous meta-analyses included case–control studies, which are insufficient to prove causal relationships due to confounding factors and reverse causality bias. In contrast, our MR results are more convincing as they avoid these confounding factors and biases. Therefore, the relationship between circadian disorders and obesity is interconnected and warrants further investigation.

The circadian rhythm and cascade of inflammation lead to the development of obesity, and the involvement of cytokines represents a complex and potential interaction (Figure 13). However, MR analysis can isolate their individual effects and evaluate the relationship between obesity and these cytokines from a genetic perspective. Despite the robustness of our findings, there are limitations to our research. Our results mainly derive from statistical analysis, and extensive experiments are necessary for future study. Genetic variation usually has a minimal impact on most risk factors, which may result in lower statistical power of MR analysis and a risk of false negative results. By multiple genetic variations related to risk factors, the proportion of variance explained can be increased, thereby enhancing statistical power. In addition, individuals of European ancestry can increase demographic bias and it may restrict the generalizability of our findings to other populations. However, the extensive genetic diversity in our datasets provides a valuable foundation for studying the correlations between circadian rhythm, immune cells, and obesity.

## 5 Conclusion

Our research findings suggest that the circadian genes (BHLHE40, CSNK1E, and PPP1CB) could serve as novel biomarkers for understanding the pathogenesis of obesity. Furthermore, we have identified potential associations between these core genes and infiltrating immune cells. These findings offer new perspectives on the prevention and treatment of obesity.

## Data Availability

Publicly available datasets were analyzed in this study. This data can be found here: GSE69039, GSE55008, GSE198012, GSE133099, and GSE155960 datasets was retrieved from GEO database.

## Footnotes

^1^ https://www.eqtlgen.org

^2^ http://app.mrbase.org/

^3^ https://gwas.mrcieu.ac.uk/

^4^ http://www.phenoscanner.medschl.cam.ac.uk/

^5^ https://go.drugbank.com/

^6^ https://www.genecards.org/
