# Supplementary figures and images for: Circadian gene signatures in the progression of obesity based on machine learning and Mendelian randomization analysis
Source: Front Nutr. 2024 Sep 16;11:1407265. doi: 10.3389/fnut.2024.1407265 (PMC11439728; doi:10.3389/fnut.2024.1407265)

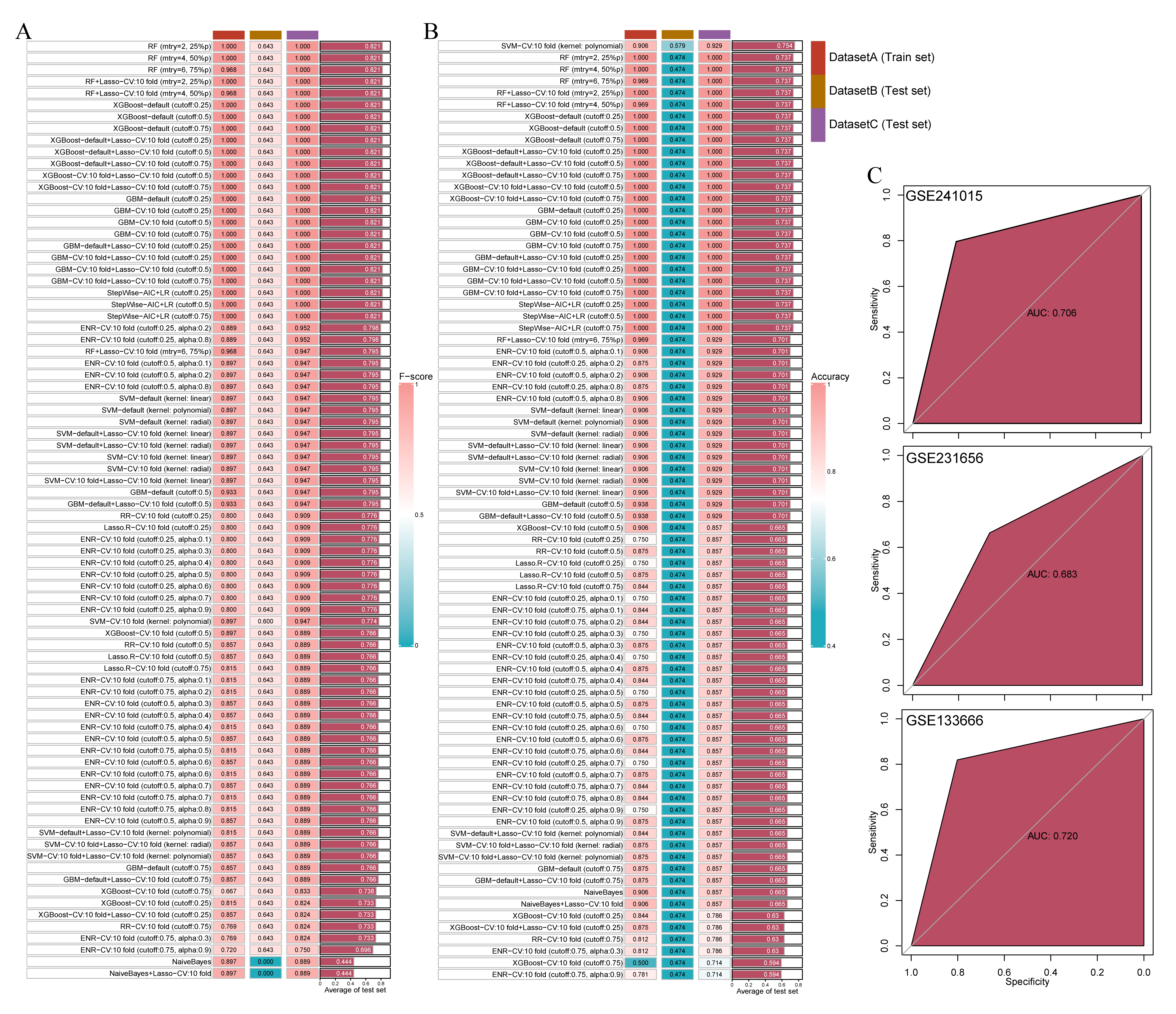

Supplement: Supplementary file 1 [file Image_1.TIF]

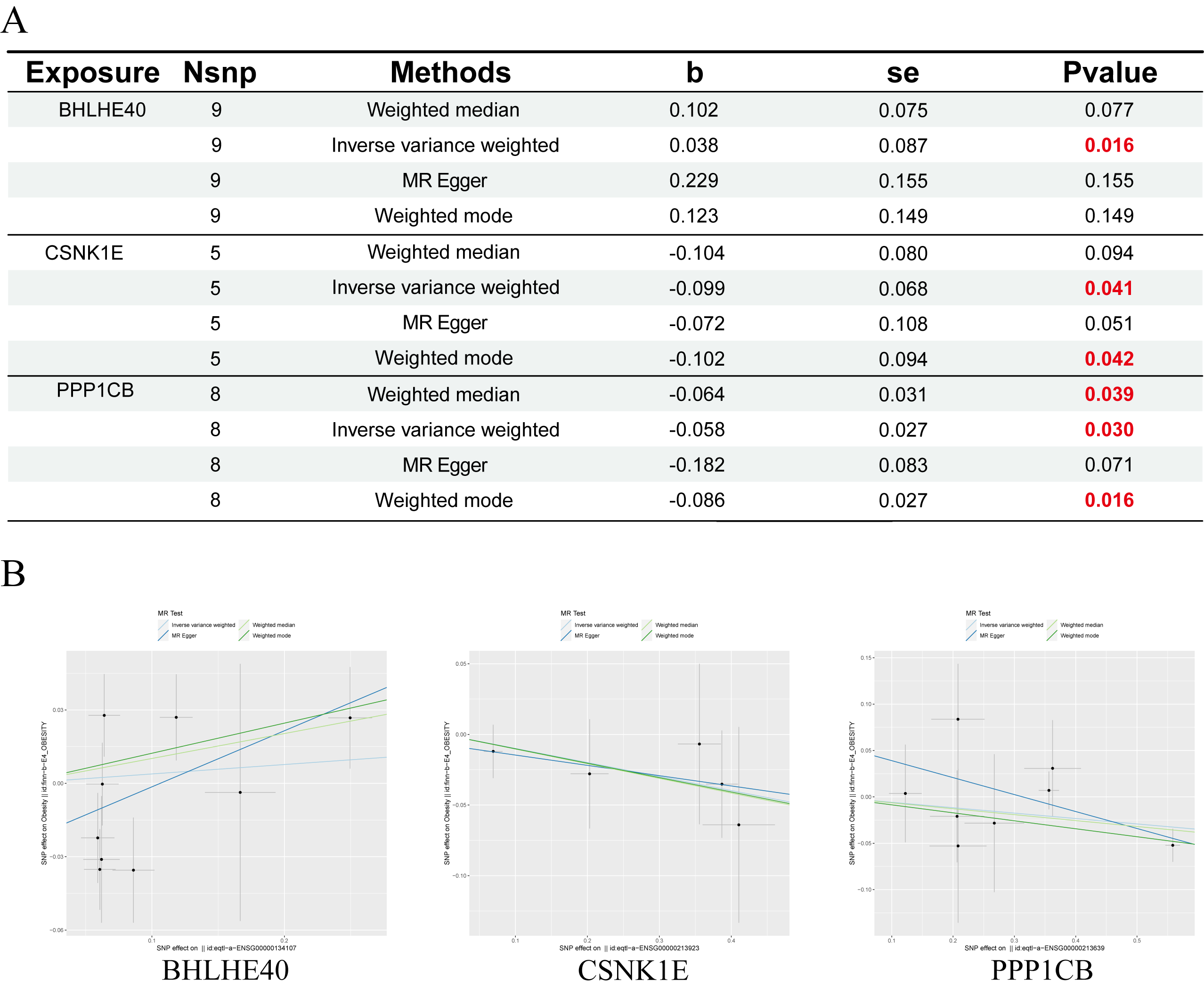

Supplement: Supplementary file 2 [file Image_2.TIF]

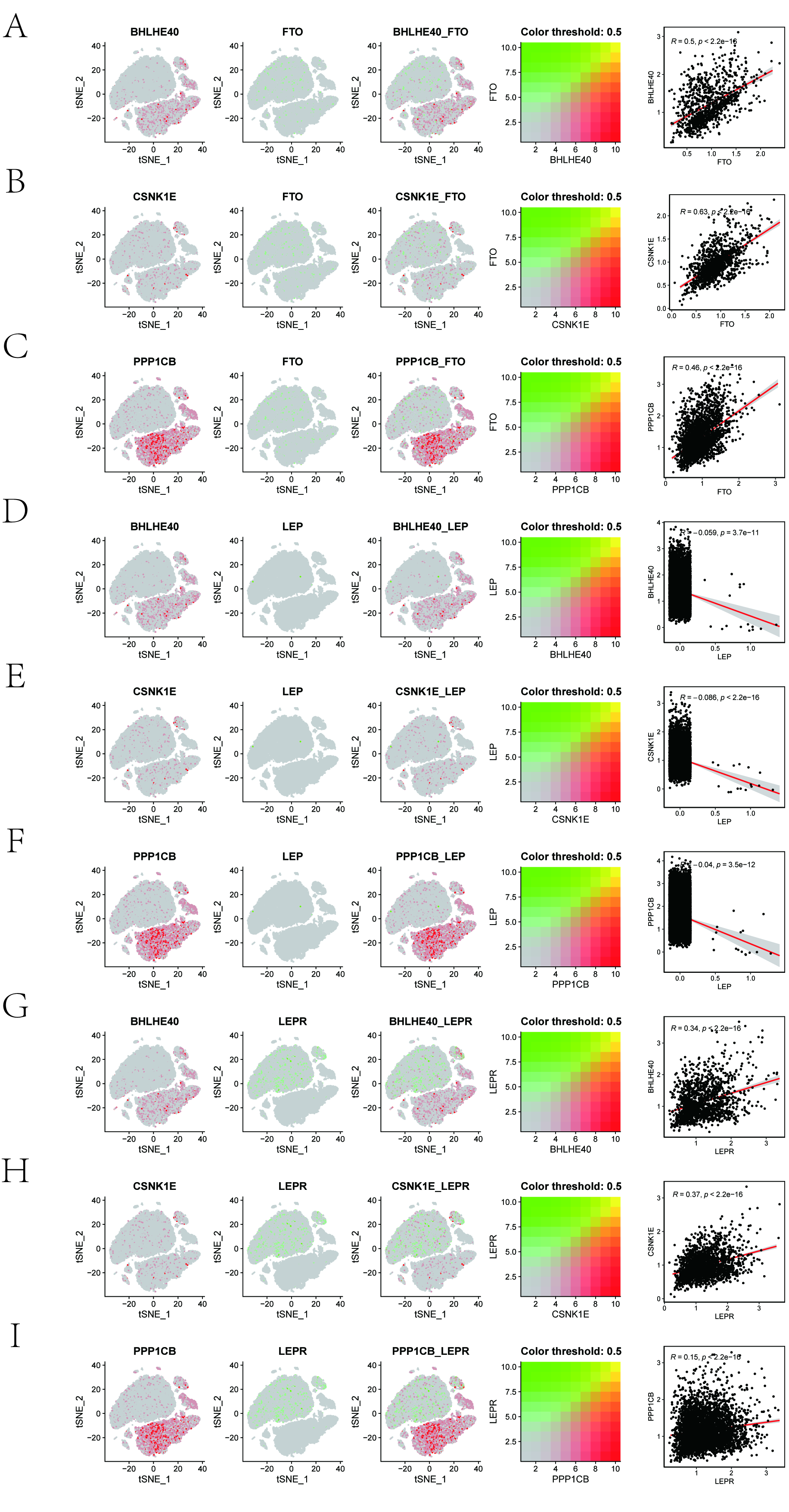

Supplement: Supplementary file 3 [file Image_3.TIF]

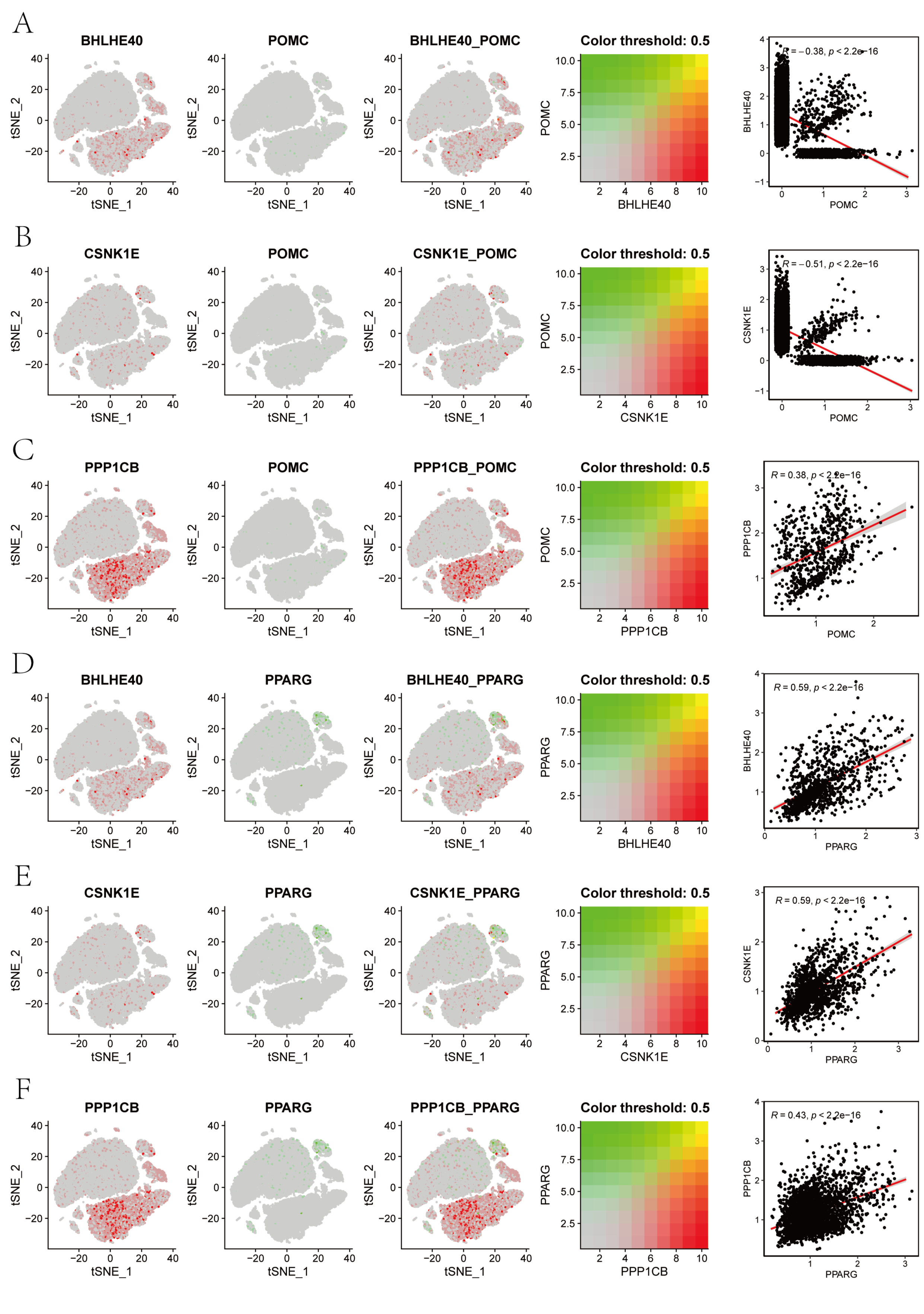

Supplement: Supplementary file 4 [file Image_4.TIF]
