# Supplementary material for: Circadian gene signatures in the progression of obesity based on machine learning and Mendelian randomization analysis
Source: Front Nutr. 2024 Sep 16;11:1407265. doi: 10.3389/fnut.2024.1407265 (PMC11439728; doi:10.3389/fnut.2024.1407265)
Supplement: Supplementary file 5 [file Image_5.pdf]

SUPPLEMENTAL MATERIAL

Circadian gene signatures in the Progression of Obesity based on Machine learning and Mendelian randomization analysis

S8.1 Animals and dietary interventions
S8.2 Glucose and insulin tolerance test
S8.3 Blood Biochemistry Test
S8.4 RNA extraction and qRT-PCR

All animal experimental procedures were conducted in accordance with the guidelines set forth by the National Institutes of Health (NIH) and were approved by the Animal Ethics Committee of Jinan University, China. The study was also carried out in compliance with the principles of the Helsinki Declaration (No. 2024-032).

S8.1 Animals and dietary interventions

Six-week-old male C57BL/6 mice were purchased from the Guangdong Medical Laboratory Animal Center in China. A total of 18 mice were included in this study. Throughout the experiment, the mice were maintained in a specific pathogen-free (SPF) environment with controlled temperature and humidity, under a 12-hour light-dark cycle, and had free access to food and water. After a one-week acclimation period, all mice were randomly divided into two groups, with 9 mice in each group. The normal chow (NC) group mice were provided with standard NC with 9% kcal fat (Trophic Animal Feed High-Tech Co., Ltd., Nantong, China) for 18 weeks. The high-fat diet (HFD) group mice were provided with HFD with 60% kcal fat (Trophic Animal Feed High-Tech Co., Ltd., Nantong, China) for 18 weeks. Body weight was measured every four weeks, including at the beginning and end of the study. Epididymal, mesenteric, perirenal, and retroperitoneal adipose tissues were collected for further experiments. All mice were euthanized by cervical dislocation after inhalation of ether.

S8.2 Blood glucose and tolerance test

For glucose tolerance test, mice were fasted overnight and then received an intraperitoneal (i.p.) injection of glucose (1 g/kg body weight). Blood glucose concentrations (tail vein) were measured via glucometer (OneTouch) at time points shown.

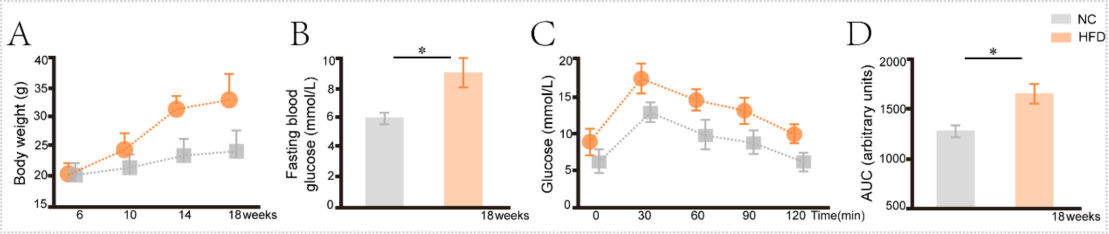

Figure S8.2 Changes in body weight and Plasma glucose in NC- and HFD-fed mice. C57BL/6 mice were fed continually for 18 weeks. **A,** Body weight changes at indicated ages (n=9). **B,** Fasting blood glucose level (n=9). **C and D,** Plasma glucose levels during GTT (n=9). Statistical analysis was performed using Student's t test. Data are expressed as mean ± standard deviation. * $P < 0.05$.

S8.3 Blood biochemistry test

Blood was collected from all 18-week-old mice in each group and gently mixed by inversion. Subsequently, the cells were allowed to stand at room temperature for 30 min to confirm blood coagulation and centrifuged at 1500 ×*g* for 20 min to obtain the serum, which was placed in a separate tube and rapidly frozen. The appropriate test kits (Wako Pure Chemical Industries Ltd., Osaka, Japan) were used to determine the levels of total lipid, LDL cholesterol, HDL cholesterol, and TG (neutral fat).

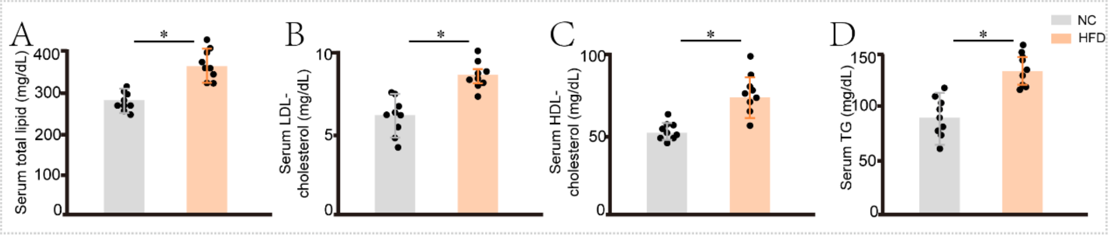

Figure S8.3 Determination of blood chemistry values in NC- and HFD-fed mice. A, total lipid. **B,** low-density lipoprotein (LDL) cholesterol. **C,** high-density lipoprotein (HDL) cholesterol. **D,** triglyceride (TG; neutral fat). n=9 per group. Statistical analysis was performed using Student's t test. Data are expressed as mean ± standard deviation. * $P < 0.05$.

S8.4 RNA extraction and qRT-PCR

Total RNA was extracted from tissues with the use of TRIzol reagent (D9108A, Takara Bio, Japan). RNA was reverse-transcribed using the RNA PCR Kit (RR036A, Takara Bio, Japan). Quantitative polymerase chain reaction (qPCR) amplification was performed using 7900HT Sequence Detection System (Applied Biosystem, Foster City, USA) using the manufacturer's protocol as recently described. Relative gene expression (normalized to endogenous control gene β-actin) was calculated using the comparative Ct method formula $2^{-\Delta\Delta Ct}$. The real-time PCR primer sequences are shown as following table1.

table1. *qRT-PCR primers used*

Gene	Forward primer	Tm	Reverse primer	Tm
β-Actin	ATATCGCTGCGCTGGTCGTC	*57.5*	AGGATGGCGTGAGGGAGAGC	*57.8*
Ppp1cb	GATGTCGTCCAGGAAAGATTGT	*60.0*	TCAGTGGTGCTTCCAATTCCA	*61.6*
Csnk1e	AAGCTCGAATGTGTGAAGACG	*60.6*	TGACCATCACGTTATAGTCTCCC	*60.9*
Bhlhe40	ACGGAGACCTGTCAGGGATG	*62.8*	GGCAGTTTGTAAGTTTCCTTGC	*60.2*
Fto	TTCATGCTGGATGACCTCAATG	*60.4*	GCCAACTGACAGCGTTCTAAG	*61.2*
Lepr	GAATGAGCAAGGTCAAAACTGC	*60.3*	CCTAGCTGGCGAAAAACTGAAG	*61.5*
Pomc	ATGCCGAGATTCTGCTACAGT	*61.0*	TCCAGCGAGAGGTCGAGTTT	*62.8*
