# Supplementary material for: Circadian gene signatures in the progression of obesity based on machine learning and Mendelian randomization analysis
Source: Front Nutr. 2024 Sep 16;11:1407265. doi: 10.3389/fnut.2024.1407265 (PMC11439728; doi:10.3389/fnut.2024.1407265)
Supplement: Supplementary file 8 [file Table_3.docx]

**Supplementary 3.** Detailed information for the exposure and outcome in MR analysis

| Gene(exposure) | Method | Obesity(outcome) | | | | | |
| --- | --- | --- | --- | --- | --- | --- | --- |
|  |  | Beta | se | *P* | *OR* | *OR_lci95* | *OR_uci95* |
| BHLHE40 | Inverse variance weighted | 0.088965 | 0.040391 | 0.027625 | 1.093042 | 1.009846 | 1.183092 |
| BHLHE40 | MR Egger | 0.044169 | 0.129313 | 0.790459 | 1.045159 | 0.811165 | 1.346653 |
| BHLHE40 | Weighted median | 0.079846 | 0.043195 | 0.064531 | 1.083121 | 0.995195 | 1.178814 |
| BHLHE40 | Weighted mode | 0.077282 | 0.045842 | 0.233873 | 1.080347 | 0.98751 | 1.181911 |
| CSNK1E | Inverse variance weighted | -0.0807 | 0.039868 | 0.042953 | 0.92247 | 0.853131 | 0.997445 |
| CSNK1E | MR Egger | -0.04026 | 0.15438 | 0.818639 | 0.960536 | 0.709745 | 1.299945 |
| CSNK1E | Weighted median | -0.09161 | 0.048306 | 0.057888 | 0.912457 | 0.830029 | 1.00307 |
| CSNK1E | Weighted mode | -0.03014 | 0.053722 | 0.614005 | 0.970315 | 0.87334 | 1.078056 |
| PPP1CB | Inverse variance weighted | -0.04317 | 0.019502 | 0.026837 | 0.957744 | 0.921827 | 0.995061 |
| PPP1CB | MR Egger | -0.16354 | 0.074683 | 0.272712 | 0.84913 | 0.733505 | 0.982982 |
| PPP1CB | Weighted median | -0.0546 | 0.017068 | 0.001379 | 0.946865 | 0.915714 | 0.979076 |
| PPP1CB | Weighted mode | -0.05816 | 0.018599 | 0.088864 | 0.943503 | 0.909728 | 0.978533 |
